# Adhesion strength of tumor cells predicts metastatic disease *in vivo*

**DOI:** 10.1016/j.celrep.2025.115359

**Published:** 2025-03-05

**Authors:** Madison A. Kane, Katherine G. Birmingham, Benjamin Yeoman, Neal Patel, Hayley Sperinde, Thomas G. Molley, Pranjali Beri, Jeremy Tuler, Aditya Kumar, Sarah Klein, Somaye Zare, Anne Wallace, Parag Katira, Adam J. Engler

**Affiliations:** 1 Chien-Lay Department of Bioengineering, UC San Diego, La Jolla, CA 92093, USA; 2 Department of Pathology, UC San Diego, La Jolla, CA 92093, USA; 3 Department of Surgery, UC San Diego, La Jolla, CA 92093, USA; 4 Moores Cancer Center, UC San Diego, La Jolla, CA 92093, USA; 5 Department of Mechanical Engineering, San Diego State University, San Diego, CA 92182, USA; 6 Computational Science Research Center, San Diego State University, San Diego, CA 92182, USA; 7 Sanford Consortium for Regenerative Medicine, La Jolla, CA 92037, USA; 8 Present address: BD Bioscience, La Jolla, CA 92121, USA; 9 Present address: Altos Labs, La Jolla, CA 92121, USA; 10 Present address: Vertex Pharmaceuticals, La Jolla, CA 92121, USA; 11 These authors contributed equally; 12 Lead contact

## Abstract

Although only a fraction of tumor cells contribute to metastatic disease, no prognostic biomarkers currently exist to identify these cells. We show that a physical marker—adhesion strength—predicts metastatic potential in a mouse breast cancer model and that it may stratify human disease. Cells disseminating from murine mammary tumors are weakly adherent, and, when pre-sorted by adhesion, primary tumors created from strongly adherent cells exhibit fewer lung metastases than weakly adherent cells do. We demonstrate that admixed cancer lines can be separated by label-free adhesive signatures. When applied to murine metastatic tumors, adhesion retrospectively predicts metastatic disease with 100% specificity, 85% sensitivity, and area under the curve (AUC) of 0.94. Cells from human reduction mammoplasties have a higher adhesion strength versus resected human tumors, which may also be stratified between invasive and more indolent cancers. Thus, highly metastatic cells may have a distinct physical phenotype that may be a predictive marker of clinical outcomes.

## In brief

Kane et al. show that adhesion measurements of cancer cells can be used to retrospectively predict metastatic capacity within an *in vivo* murine model. Additionally, adhesion can be used to stratify human patient samples into healthy reduction tissue, non-invasive ductal carcinoma *in situ*, and invasive ductal or lobular carcinoma.

## Graphical Abstract

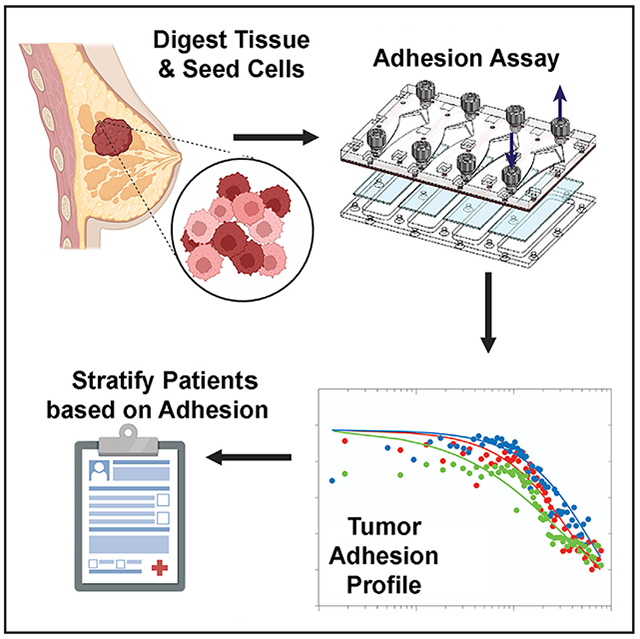

## INTRODUCTION

Tumors are often detected and treated when disease is local, but, when epithelial carcinomas become regionally invasive, 5-year recurrence rates can exceed 15% for breast cancer and are even worse for other solid tumors.^1,2^ Patient relapse is multifactorial, but it is due in part to our inability to identify metastasizing cells early, quantify their presence in stroma, and create appropriate risk assessments to guide standards of care. Significant efforts have attempted to identify universal molecular prognostic markers^3,4^ from liquid biopsies but have only identified tumor-specific markers at best,^3,5^ owing in part to cell heterogeneity^6^ and a lack of marker exclusivity.^3,7,8^ These assays also only surveil cells post intravasation, missing an opportunity to assess dormant cell populations resident in tumor-adjacent stroma.^9^

Metastasis can be compartmentalized into a series of discrete physical events required for all solid tumors: detachment from tumor, migration through stroma, and intravasation into the blood stream.^10–13^ At each step, cells undergo numerous, distinct biophysical changes that enable metastasis.^11,14–18^ As with many molecular marker assays, recent US Food and Drug Administration (FDA)-approved devices that utilize biophysical markers focus on detecting circulating tumor cells (CTCs). However, disease may have progressed too far by the time CTCs become detectable in the blood, reducing the effectiveness of their prognostic assessment; tumor cells can often remain dormant in stroma for years,^9^ and significant sample dilution may allow some CTCs to escape detection. Conversely, physical assays that interrogate cells in the stroma are now being used to probe cells from these heterogeneous yet dormant populations^11^ and determine their mechanotypes.^19^ For example, microchannel assays that confine cancer cells as they migrate have been used to determine invasive mechanisms^20^ and to assess progression-free survival,^21^ making widespread clinical adoption more feasible. Weak adhesion strength is another biophysical metastatic marker that promotes migration via increased focal adhesion turnover.^22–29^ Patients with triple-negative breast cancer whose tumors have transcriptomic profiles mirroring weakly adherent cells also have shorter disease-free intervals,^29^ suggesting a correlation between clinical outcomes and physical properties (i.e., an adhesion mechanical phenotype or “mechanotype”).^30^

Despite strong evidence, the predictive capacity of adhesion for metastatic disease is not clear. Herein, we use straight- and divergent-wall parallel-plate flow chambers (PPFCs) to evaluate sorting and analysis capabilities using mouse models, admixed cell populations, and human tumors. We find an inverse relationship between adhesion strength and metastatic behavior using a murine mammary tumor model. Cells that sort as weakly adherent are more migratory, result in more secondary disease, and have a stable mechanotype *in vivo*. PPFC analyses provide label-free measurements of adhesion strength and cell abundance relative to the analyzed population, which when used for admixed cancer lines enables separation and when applied to metastatic disease can retrospectively predict metastatic potential in a mouse model. We also observed an inverse relationship between adhesion strength and metastatic capacity for human cancers versus reduction mammoplasties; cells from patients with invasive cancer are more weakly adherent than cells from non-invasive cancer or reduction mammoplasties. These results suggest that cells with a more weakly adherent phenotype migrate out of the primary tumor microenvironment and can be isolated and quantified by this device in a manner that could provide a prognostic tool to assess future patient outcomes.

## RESULTS

### Invading cells have decreased adhesion strength compared to primary tumors in a murine model

**Figure 1.**
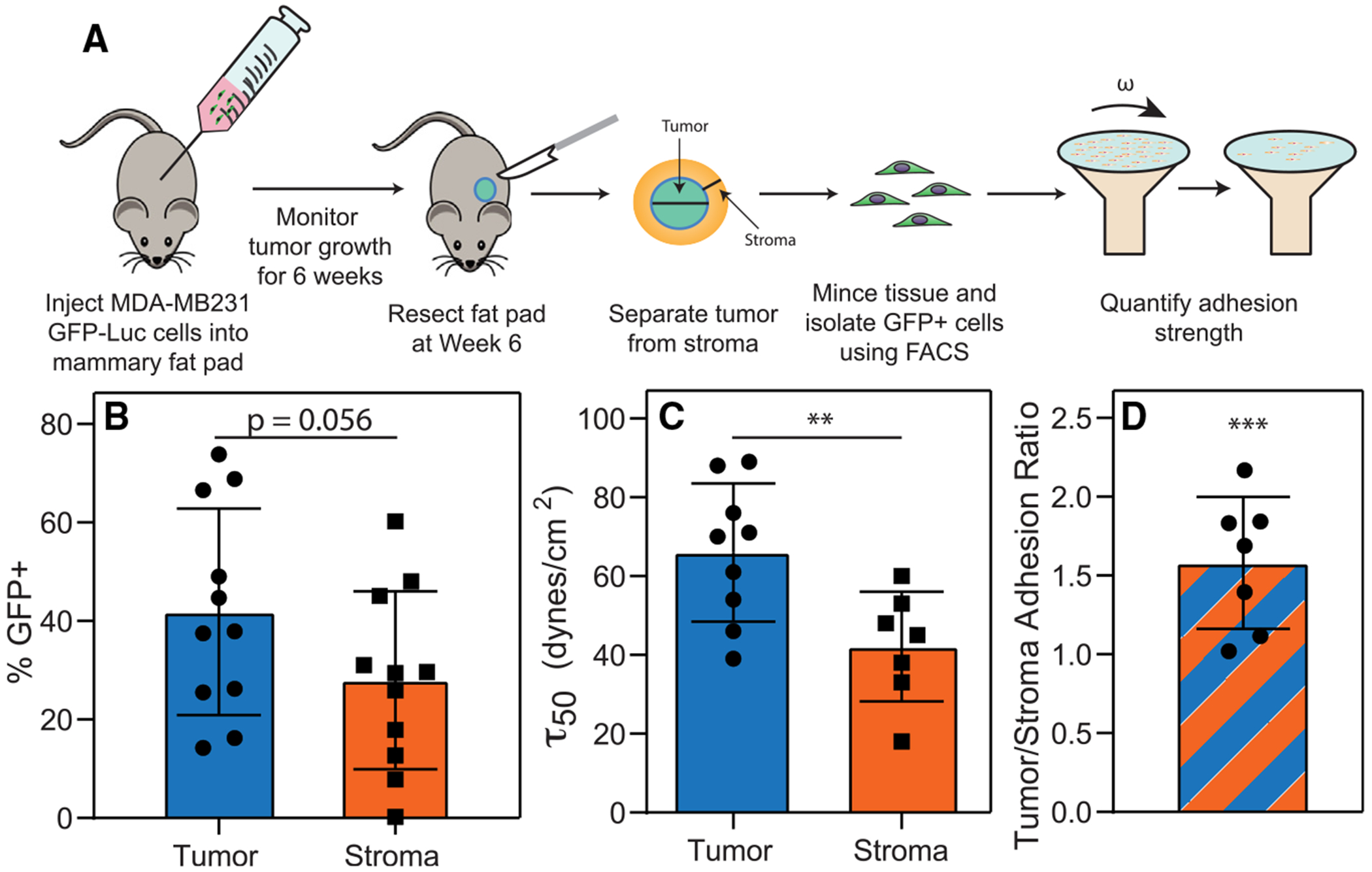
MDA-MB231 cells that have invaded into the fat pad display decreased adhesion strength compared to tumor cells (A) Timeline of tumor resection and adhesion strength study. (B) There are fewer GFP-positive cells present in the stroma versus the tumor (*n* = 11). (C and D) Invaded GFP^+^LUC^+^MDA-MB231 cells have decreased adhesion strength compared to MDA-MB231 cells that remain in the tumor (*n* = 9 and 7 for tumor and stroma, respectively; *n* = 7 for paired samples in D). Tumor/fat pad adhesion ratio is the $\tau_{50}$ of tumor cells divided by the $\tau_{50}$ of cells derived from the surrounding fat pad. (B–D) Statistical analysis via unpaired t test. **p < 0.01 and ***p < 0.001. For (D), statistical comparison for paired samples was assessed relative to the null hypothesis. Bar graphs and error bars in (B)–(D) are expressed as mean ± standard deviation.

Metastatic cell lines have lower adhesion strength and more labile focal adhesions than their non-metastatic counterparts.^28^ To determine to what extent a mouse tumor model phenocopies adhesion, MDA-MB231 human breast carcinoma cells were exposed to lentiviral vectors containing GFP and firefly luciferase (Luc) and selected for both markers (Figures S1A and S1B). To ensure that transduction did not impact adhesion strength and migration, cells were selectively exposed to pre-determined shear stresses to isolate weakly and strongly adherent fractions,^11^ and their migration was assessed on collagen gels; weakly adherent cells exhibited greater migration speed and displacement compared to strongly adherent cells and independent of transfection (Figures S1C and S1D), indicating that GFP and Luc transduction had minimal impact on function. Given that adhesion and migration speed are inversely correlated *in vitro*, we next determined this relationship *in vivo*. GFP^+^Luc^+^ MDA-MB231 cells were injected into inguinal fat pads of 11-week-old NOD/SCIDγ (NSG) mice, monitored at 2-week intervals, and resected at 6 weeks post injection (Figures S2A and S2B). GFP^+^ cells were isolated from tumor and surrounding fat pad, sorted using fluorescence-activated cell sorting (FACS), and their adhesion profile quantified (Figures 1A and S2C). The GFP^+^ fraction in the fat pad was lower than in tumor (Figure 1B) and significantly less adhesive compared to tumor-resident GFP^+^ cells (Figure 1C). For paired samples isolated from the same fat pad, GFP^+^ tumor cells were 60% more adherent than GFP^+^ fat pad cells (Figure 1D), suggesting that cells of the weakly adherent mechanotype escape the tumor, consistent with the observations in culture.^30^

### Cell-intrinsic adhesion differences correlate with lung metastatic frequency in murine models

**Figure 2.**
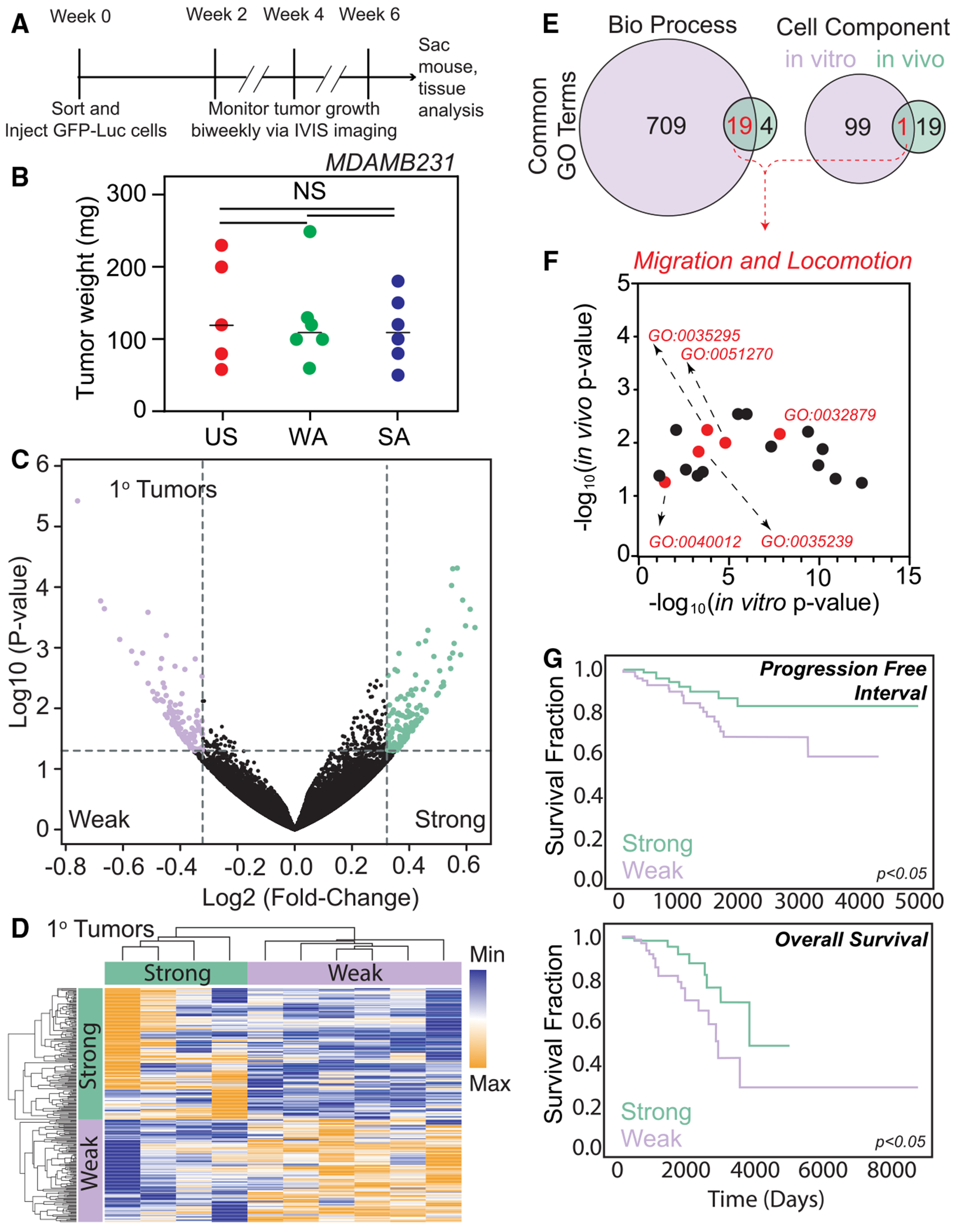
Primary tumors cluster based on adhesion phenotype of injected cells despite no difference in primary tumor size among groups (A) Timeline of the lung metastases tumor study. (B) Tumor weight at time of sacrifice 6 -weeks post injection. *n* = 5, 6, and 6 for mice with unsorted (US), weakly adherent (WA), and strongly adherent (SA) MDAMB231 cells injected, respectively. (C) Differences in gene expression between WA and SA primary tumors are shown in a volcano plot (*n* = 265 DEGs). Data represent four SA and six WA tumors; two additional SA tumors were omitted for low RNA quality. (D) Hierarchical clustering of DEGs between WA and SA cells. Vertical bars indicate clustering of genes that are upregulated in SA cells and WA cells (*n* = 4 SA and 6 WA tumors). (E) Venn diagram of Gene Ontology (GO) terms associated with DEGs. Numbers indicate the number of terms present in each group. (F) GO terms that are upregulated in WA tumors. Red terms indicate association with cell migration or locomotion. (G) TCGA analysis of the expression levels of genes associated with regulation of locomotion in 112 patients with estrogen receptor (ER)–, progester-one receptor (PR)–, and Her2– breast cancer. Statistical analysis by (B) one-way ANOVA with Tukey test for multiple comparisons and (G) log-rank test.

While cells that disseminate are less adherent, it is not clear whether this translates to a greater propensity to form metastatic tumors at distant sites. To artificially create differences in metastatic potential between tumors, GFP^+^Luc^+^ MDA-MB231 cells were first sorted by adhesion using a PPFC (Figure S3) and then injected into inguinal fat pads. Tumors were allowed to grow for 6 weeks prior to assessing primary tumors and secondary disease in the lungs (Figure 2A). While there was no difference in primary tumor weight (Figure 2B), bioluminescent imaging area, or flux (Figures S4A and S4B), transcriptional analyses of primary tumors found 265 differentially expressed genes (DEGs) associated with adhesion strength (Table S1; Figures 2C and 2D), indicating that they may retain pre-injection differences. Gene Ontology (GO) terms associated with DEGs were stratified and compared to those of cells sorted *in vitro* (Figure 2E; Table S2)^29^; we found significant GO term overlap for biological processes, and, among these terms, many were associated with cell migration and locomotion (Figure 2F, red; Table S3). Further, The Cancer Genome Atlas (TCGA) analysis revealed that, among the genes regulating locomotion (GO:0032879; Table S4), high expression is significantly correlated with poorer patient prognosis and progression-free interval (Figure 2G). These data suggest that a common gene signature may correlate with metastatic risk and suggest what constitutes an adhesive mechanotype.

**Figure 3.**
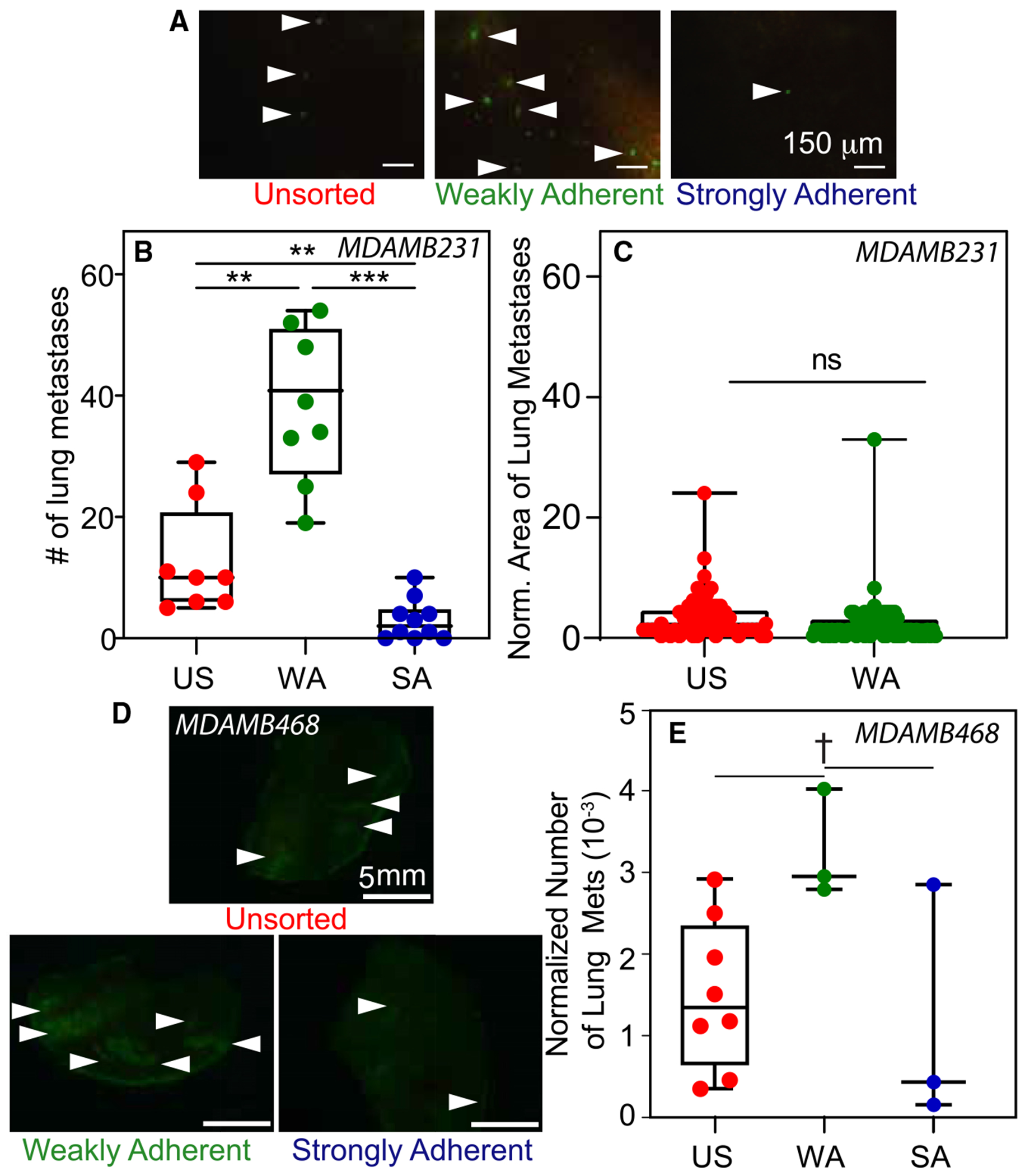
Mice injected with WA cells have more lung metastases (A) Representative images of whole, resected lungs with GFP^+^ metastases from unsorted (US), weakly adherent (WA), or strongly adherent (SA) MDA-MB231 cells (left, middle, and right column, respectively). Scale bar, 150 μm. Arrowheads indicate metastases. (B) Plot of the number of lung metastases in mice injected with US, WA, or SA MDA-MB231 cells (*n* = 8, 8, and 10 for primary tumors composed of US, WA, and SA cells, respectively). (C) Plot of the size of lung metastases (*n* = 57 and 78 lesions analyzed for US and WA, respectively), excluding SA tumors given the low frequency of lesion formation. (D) Representative images of lung metastases from US, WA, or SA MDA-MB468 cells (top, bottom left, and bottom right, respectively). Scale bar, 5 mm. Arrowheads indicate metastases. (E) The number of lung metastases in mice injected with US, WA, or SA MDA-MB468 cells (*n* = 8, 3, and 3 for tumors composed of unsorted, WA, and SA cells, respectively) was normalized by the number of cells injected and plotted. (B, C, and E) Statistical analysis via (B and E) one-way ANOVA with Tukey test for multiple comparisons or (C) unpaired t test. ^†^*p* < 0.1, **p < 0.01, and ***p < 0.001. ns, no significance. (B, C, and E) Box-and-whisker plots show boxes indicating the 25th to 75th percentiles, mean as a line within the box, and whiskers indicating the minimum and maximum spread of the data.

To directly assess metastatic burden, we determined the number of GFP^+^ lesions from human MDA-MB231 cells in resected mouse lungs. Mice receiving weakly adherent cells exhibited more metastases than those injected with strongly adherent cells or the unsorted parental line (Figures 3A and 3B); mice injected with strongly adherent cells consistently showed minimal metastatic activity in stark contrast to the broad range and greater average number of metastatic lung tumors in mice that received unsorted paternal or weakly adherent populations (Figures 3B, S5A, and S5B). Metastatic tumor size was not affected by pre-sorting on adhesion strength (Figure 3C), indicating that the fraction of cells disseminating, but not secondary tumor growth, differ with pre-injection adhesion sorting. These data were confirmed in a second metastatic line (i.e., MDA-MB468 cells), which, like MDA-MB231 cells, develop significant secondary disease in the lungs.^21,31,32^ We found no differences in bioluminescent signal or tumor weight as a function of adhesion sorting (Figures S4C–S4E), but there were differences in the number of metastases detected by GFP^+^ signal in lungs when normalized to the number of injected cells (Figures 3D and 3E). Thus, differences may occur in the migration machinery of cells leaving the primary tumor; hence, tumors composed of only strongly adherent cells are less likely to metastasize.

**Figure 4.**
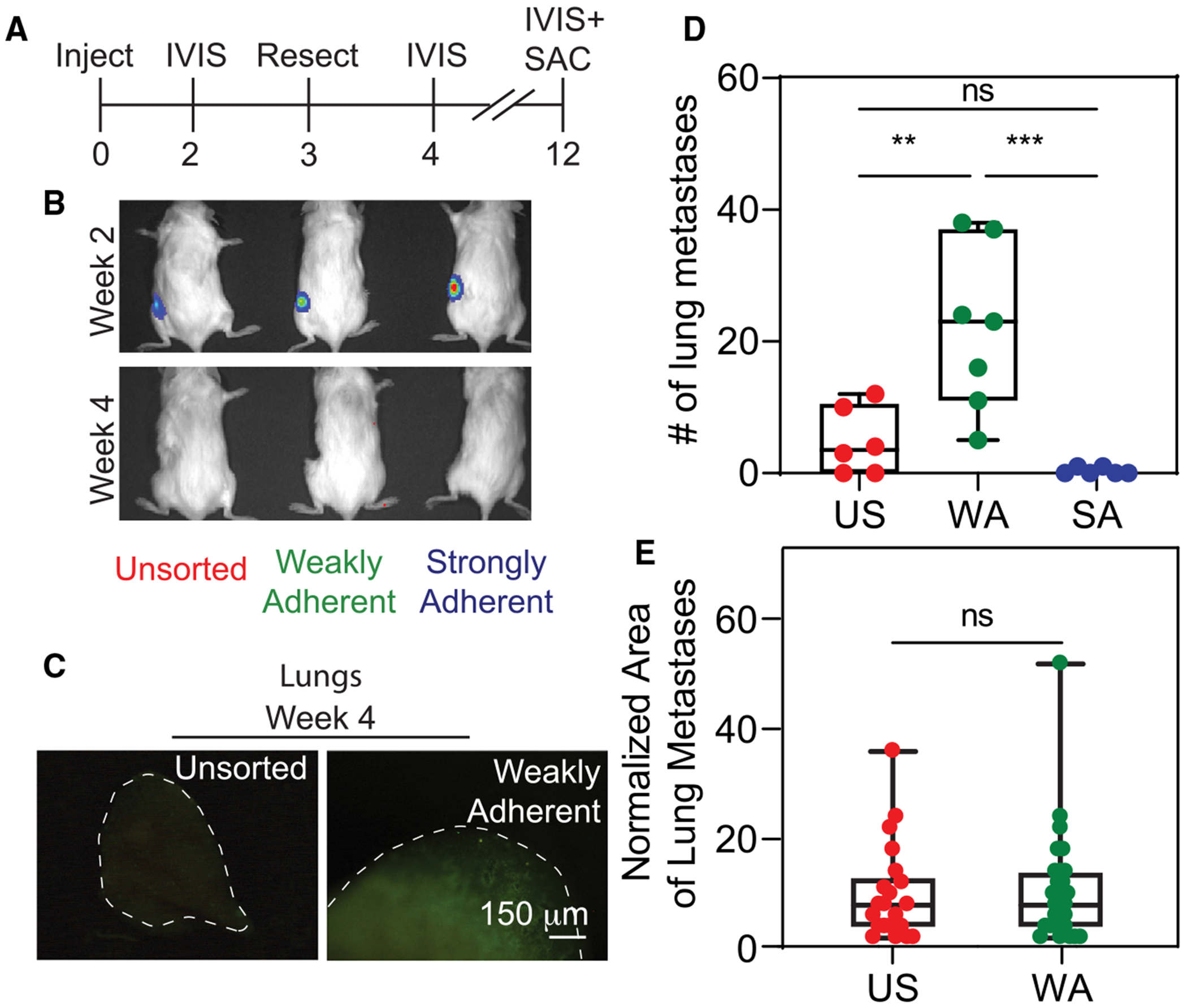
WA cells form significantly more metastases despite resection (A) Timeline of the tumor resection study using MDA-MB231 cells. (B) Bioluminescence imaging showing the developing tumors (week 2) and the post-surgery loss of signal (week 4). (C) GFP images of lungs at 4 weeks from selected mice not included in the 12-week time course. Dashed line outlines the lung lobe in the images. Scale bar, 150 μm. (D) The number of lung metastases in mice injected with unsorted (US), weakly adherent (WA), or strongly adherent (SA) MDAMB231 cells (*n* = 6, 7, and 6 for primary tumors composed of US, WA, and SA cells, respectively). (E) Size of lung metastases (*n* = 21 and 28 lesions analyzed for US and WA, respectively), excluding SA tumors given the low frequency of lesion formation. Statistical analyses were performed via (D) one-way ANOVA with Tukey test for multiple comparisons or (E) unpaired t test. **p < 0.01 and ***p < 0.001. ns, no significance. (D and E) Box-and-whisker plots show boxes indicating the 25th to 75th percentiles, mean as a line within the box, and whiskers indicating the minimum and maximum spread of the data.

While a 6-week mouse model showed significant differences for weakly adherent tumors, standard of care typically involves tumor resection. As such, we included tumor resection at 3 weeks post injection (Figures 4A and 4B), which was the time point when tumors were first manually palpable but the lungs were still largely clear of nodules (Figure 4C). After confirming the presence of, and then absence of, primary tumors based on luminescent signal pre- and post resection at week 3 (Figure 4B), respectively, we assessed the lung tumor burden at 12-week post injection. Despite removal of the tumors (confirmed by gross anatomical inspection, not shown), we found that lung tumor burden at this time point was significantly higher for weakly adherent cells versus unsorted cells; for strongly adherent tumors, only one-third produced detectable metastases and only one per mouse (Figures 4D and S5C). Again, metastatic tumor size was not affected by pre-sorting on adhesion strength (Figure 4E), despite the resection. These data indicate that significantly more weakly adherent cells escape the tumor early and then metastasize and form secondary disease after resection of the primary tumor.

### Differences in metastatic and non-metastatic adhesion strength sort admixed populations

**Figure 5.**
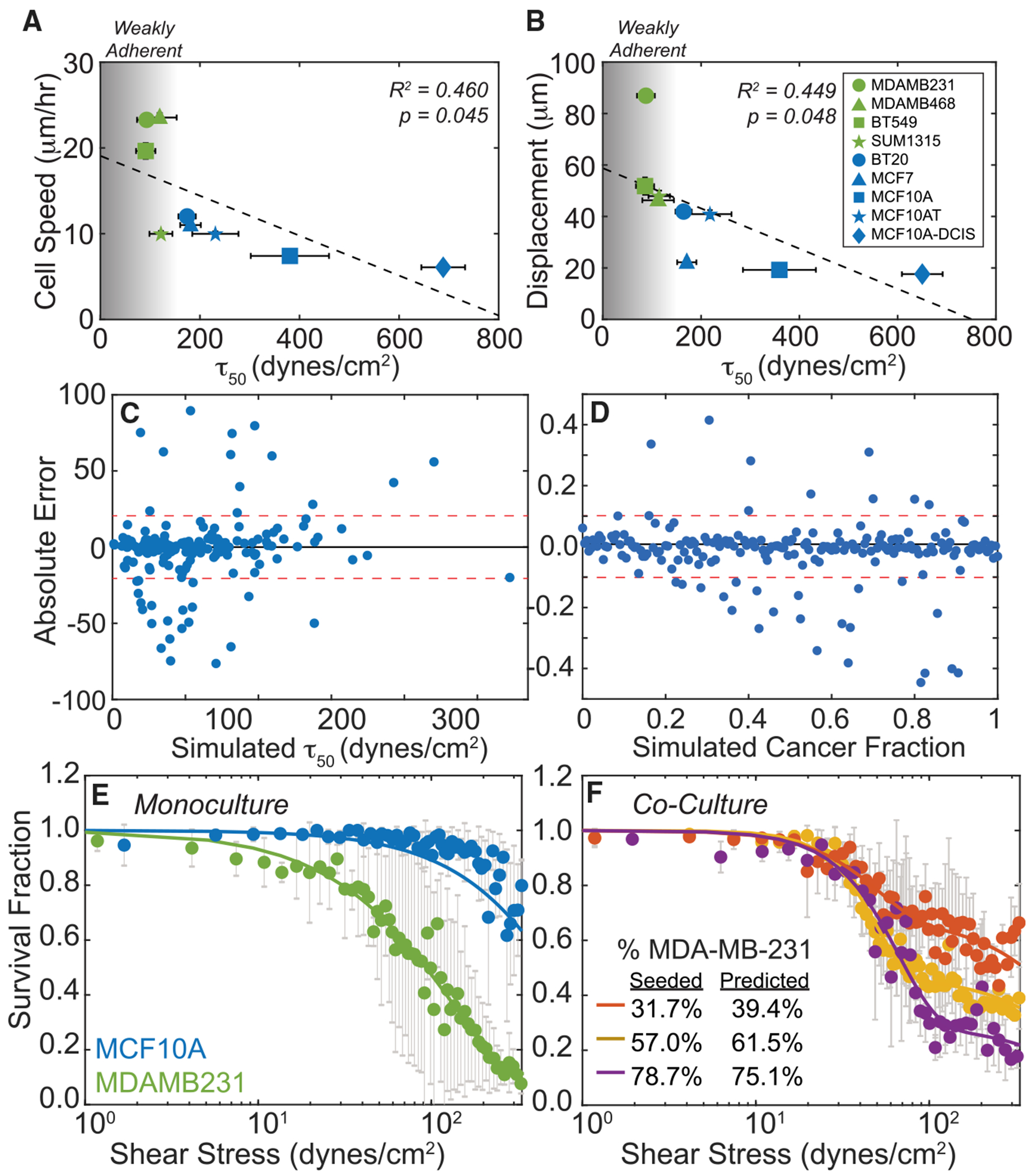
Cell-line metastatic potential correlates with a decreased adhesion strength (A) Cell speed and (B) displacement versus average detachment shear stress from fibronectin for various metastatic (green) and non-metastatic cell lines (blue). Gradient represents shift from WA cells (gray) to SA cells (white). For cell speed and displacement, metastatic cell lines (green) had *n* = 553, 475, 137, and 306 cells analyzed for MDA-MB231, MDA-MB468, BT459, and SUM1315, respectively. For cell speed and displacement, non-metastatic cell lines (blue) had *n* = 609, 253, 253, 225, and 305 cells analyzed for BT20, MCF7, MCF10A, MCF10AT, and MCF10AT-DCIS, respectively. For adhesion strength, metastatic cell lines (green) had 13, 10, 13, and 12 replicates for MDA-MB231, MDA-MB468, BT459, and SUM1315, respectively. For adhesion strength, non-metastatic cell lines (blue) had 12, 10, 8, 9 , and 9 replicates for BT20, MCF7, MCF10A, MCF10AT, and MCF10AT-DCIS, respectively. y axis error bars denote the standard deviation and x axis error bars denote the standard error of the mean. (C and D) (C) The absolute error of simulated average shear stresses (*n* = 192) and (D) simulated cancer fractions (*n* = 196). (E) Shear stress plots of monocultured MCF10A (blue) and monocultured MDA-MB231 (green) cells calculated using a dPPFC. (F) Shear stress plots of cocultured MCF10A and MDA-MB231 cells (*n* > 3 replicates). (E and F) Error bars denote the standard error of mean.

To determine whether the inverse relationship between adhesion strength and metastatic propensity is not specific to MDA-MB231 or MDA-MB468 cells, adhesion strength of seven other breast cancer and epithelial cell lines was measured using a divergent PPFC (dPPFC) where shear stress scales with chamber position (Figures S6A–S6D and S7). For each cell line, average adhesion strength on fibronectin-coated chambers was plotted against migration speed and displacement on collagen hydrogels matched to breast-tumor stiffness.^33^ Cell lines that detached at <150 dyn/cm^2^ have been largely characterized as metastatic,^21,31,32^ whereas those detaching at >150 dyn/cm^2^ were derived from primary tumor or non-cancerous lines; thus, lower adhesion strength had a strong correlation with an increase in cell speed and displacement (Figures 5A and 5B), suggesting that an inverse relationship between adhesion strength and migration is not unique and that the dPPFC can measure parameters potentially important to patient survival outcomes. We further confirmed that the ligand used in the dPPFC did not impact outcomes; when grown on collagen-coated chambers, we also observed a negative relationship between collagen-based adhesion strength and cell speed and displacement on collagen matrices (Figure S8). While these data exhibit more noise relative to fibronectin-coated devices, at least they imply common behavior between integrin receptors.

Since mouse and patient tumors are likely a mixed population of metastatic and non-metastatic cells, we developed a deconvolution method to distinguish adhesion profiles of two populations with different adhesion strengths. Using the parameters of a Weibull distribution to describe MDA-MB231 and MCF10A adhesion profiles (see STAR Methods and Equation 3), we simulated mixtures with varying adhesion strength to test the accuracy of the method’s predictions of T_50_ and cancer cell fraction in a virtual mixture. For monocultures, simulated populations randomly selected from Weibull distributions of MDA-MB231 and MCF10A cells created adhesion profiles that matched experimental observations (Figure S6E). For co-cultures simulated with pre-determined fractions of MCF10A and MDA-MB231, we used a two-population Weibull distribution to describe the overall obtained adhesion profile (see STAR Methods; Equation 6; Figure S6F) based on the adhesion profiles of non-metastatic cells (e.g., MCF10A). We predicted the adhesion strength and metastatic cell fraction (e.g., MDA-MB-231) in the mixture and found that 80% of predictions for the metastatic population fell within ±20 dyn/cm^2^ and that 80% of predictions of the cancer fraction (i.e., the percentage of the population that is metastatic) fell within ±0.06 of simulated values (Figures 5C and 5D). We validated mixtures experimentally by seeding MDA-MB231 and MCF10A cells at known ratios into the dPPFC and measuring their combined adhesion profiles. The T_50_ and cancer fraction were predicted using the same deconvolution method, and all cancer fractions were within ±7.7% and T_50_ values were within ±43 dyn/cm^2^ of the measured monoculture values (Figures 5E and 5F). These results suggest that the dPPFC can accurately measure the cancer cell adhesion profile for heterogeneous populations without prior sorting.

### Label-free assessment of murine stromal biopsies retrospectively predicts metastatic risk *in vivo*

To assess dPPFC functionality in predicting metastatic risk (i.e., high risk being ≥2 GFP^+^ nodules), we analyzed resected tumors, stroma, and contralateral fat pads from NSG mice. The adhesion profile of contralateral fat pads, which lack GFP^+^ cells, was used to separate out host signal from the cancer fraction and of stroma and tumor samples from the recipient fat pad (Figure S9A); cell adhesion strength from the host’s contralateral fat pads were ~3-fold higher than any of the cancer cells from the tumor or stroma samples (Figure S9B). As outlined in the STAR Methods and as used in admixed cell cultures above, we next employed a deconvolution method to distinguish adhesion profiles of host (from contralateral fat pad) vs. tumor (see Equation 6).

**Figure 6.**
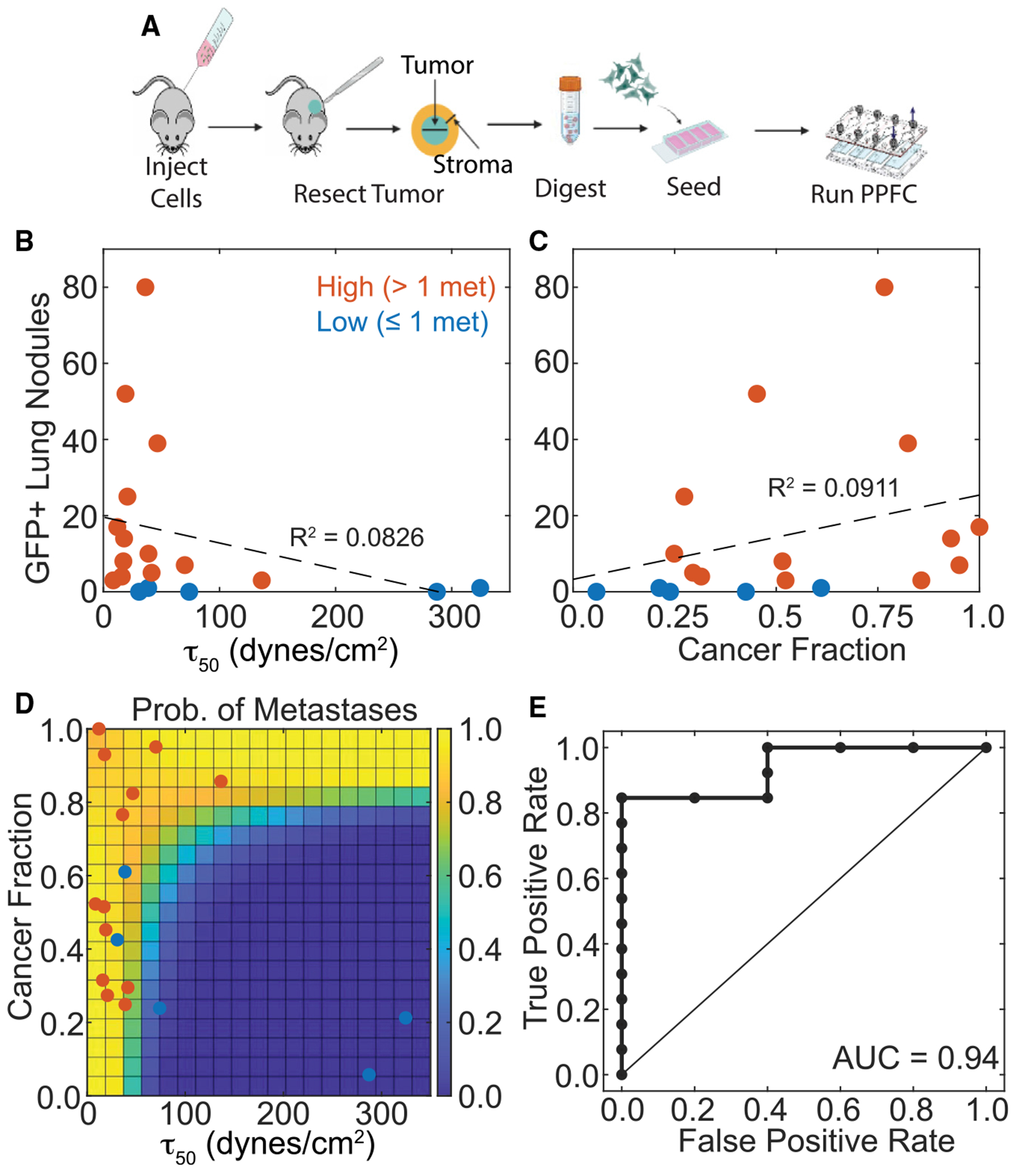
Average shear threshold of stroma surrounding the primary tumor predicts number of metastases *in vivo* Tumors and surrounding stroma were dissected from mice injected with SA, WA, or US MDA-MB-231 cells. (A) Workflow for obtaining tissue, digesting into single cell suspension, and seeding within dPPFC. (B and C) (B) The average shear stress, and cancer fraction (C), as calculated from the combined adhesion profile of GFP^+^ cancer cells and mouse cells, plotted against the number of GFP^+^ lung nodules (*n* = 18 lungs). Red points are classified as high metastatic risk (i.e., ≥2 GFP^+^ nodules) and blue are low (i.e., <2 GFP^+^ nodules). (D) Logistic regression model showing probability estimate of a mouse having ≥2 GFP^+^ metastatic lung nodules based on the average shear stress and cancer fraction measured from the dPPFC. (E) Receiver operating characteristics (ROC) curve of metastatic risk predictions based on model’s probability estimates. Red points are classified as high metastatic risk and blue are low. 18 paired tumor and stromal biopsies were used from different mice. Each biopsy was split into two replicates for adhesion measurements.

The number of GFP^+^ nodules observed in the lungs was inversely proportional to the T_50_ measured from the tumor and stroma samples (Figures 6B and S9C). Only in the stroma was there a positive correlation with the estimated cancer fraction of the sample with GFP^+^ lung nodules (Figure 6C), whereas, in the tumor biopsy, there was less correlation (Figure S9C). Next, we performed a receiver operating characteristic (ROC) analysis to determine prediction accuracy for the cancer fraction and T_50_ for either high or low metastatic risk, defined as high risk if ≥ 2 GFP^+^ nodules were counted in the lungs. Classification accuracy was highest from stromal cancer fraction with an area under the curve (AUC) of 0.83 (Figure S9D). However, by fitting the number of metastatic lesions, T_50_, and cancer fraction data with a logistic regression model, we generated probability estimates for ≥2 GFP^+^ nodules that proved better at predicting risk than primary tumors (Figures 6D, S9E, and S9F); the regression model for these data was more statistically different versus a constant model as determined by a deviance test (*p* = 0.0167) than for the primary tumor. We then used the probability estimates in the ROC analysis to evaluate how well the cancer fraction and its $\tau_{50}$ classify metastatic risk. Combining both metrics together improved prediction accuracy, increasing the AUC to 0.94 and specificity to 100% with a sensitivity of 85% (Figure 6E). Improved prediction in the stroma at 6 weeks versus tumor core may be due to weakly adherent cells having already disseminated from the tumor (e.g., Figure 1C); hence, stroma better captures the metastatic potential of the tumor at that point in primary tumor progression. Altogether, these data show that the dPPFC can detect cancer cells that have locally invaded the surrounding stroma, and their quantity and adhesion mechanotype can be used to predict an increased rate of metastatic tumor formation.

### Patient cells derived from invasive cancer tissue have a lower adhesion strength than cells from non-invasive cancer or healthy reduction tissue

**Figure 7.**
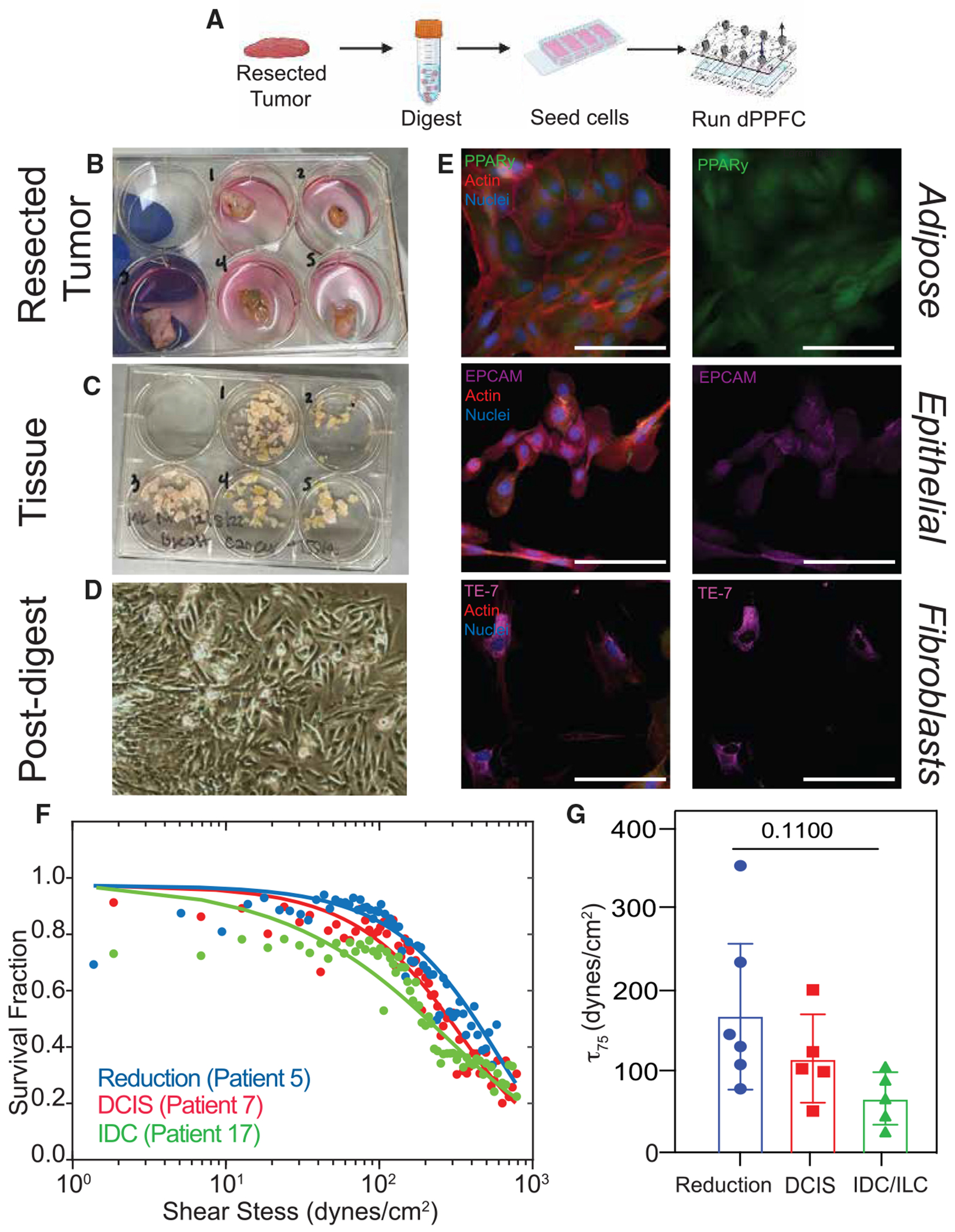
Adhesion strength of cells from lumpectomies is lower than adhesion strength of cells from reduction mammoplasties Tissue from patients undergoing reduction mammoplasties or lumpectomies were collected and digested to single-cell suspensions to run on dPPFC. (A) Timeline of patient collection and digestion. (B–D) (B) Patient tissue pre-digest, (C) digested tissue, and (D) cells seeded on tissue-culture plastic post digest. (E) Immunofluorescence images of cells from patient E009 to stain for PPARγ, EPCAM, TE-7, actin, and nuclei. Scale bar, 100 μm. (F) Adhesion curve of reduction, ductal carcinoma *in situ* (DCIS), and invasive ductal carcinoma (IDC) tissue; technical replicates from patient 5 were used to generate reduction curve, technical replicates from patient 7 were used to generate DCIS curve, and technical replicates from patient 17 were used to generate IDC curve. One-population Weibull distribution function was used to fit the data. (G) $\tau_{75}$ of reduction, DCIS, and invasive ductal carcinoma/invasive lobular carcinoma (*n* = 16 samples). Statistical analysis was performed via one-way ANOVA with Tukey test for multiple comparisons. Bar graphs and error bars are expressed as mean ± standard deviation.

To determine whether weakly adherent cells are the primary drivers of metastatic disease in human patients, we obtained tissue specimens of healthy breast from reduction mammoplasty (six samples), non-invasive cancer (ductal carcinoma *in situ* [DCIS]; five samples), and invasive lobular or ductal carcinoma (ILC or invasive ductal carcinoma [IDC]; five samples) to compare their adhesion characteristics. Upon receipt, tissue was digested to a single-cell suspension, expanded, and either immunostained or run on the dPPFC (Figures 7A–7D). All samples stained positively for adipocytes, epithelial cells, and fibroblasts (Figure 7E), making these human tumors more heterogeneous in cell composition relative to mouse tumors. The presence of multiple phenotypes likely creates an adhesion-strength continuum in human tumors versus discrete weakly or strongly adherent populations in mouse tumors. Thus, resected human tumor cells were analyzed by a one-population Weibull distribution (Figure 7F; Equation 4) and the $\tau_{75}$, which represents the adhesion strength at which 25% of cells detach from their substrate, was reported for human tissue. $\tau_{75}$ measurements were reported instead of $\tau_{50}$ as the weakly adherent population within patient tissues should be better represented by $\tau_{75}$ versus $\tau_{50}$. 95% confidence intervals were determined, and we found high R^2^ values for control mammoplasty and tumor samples (Figures S10 and S11). Hence, contributions from weakly adherent cells in patient tumors did not represent a discrete population but rather resulted in a leftward shift in the adhesion curve relative to mammoplasty controls. In examining the 16 patient samples, we found that the average $\tau_{75}$ of healthy breast tissue could be stratified by invasiveness: the overall single-cell population recovered from healthy breast tissue was most strongly adherent, cells from DCIS had intermediate average population adhesion strength, and cells obtained from IDC and ILC tumors were the most weakly adherent across patients (Figure 7G). These results are consistent with data from the murine models; weakly adherent cells *in vitro* and in murine models are more migratory. Thus, we infer that the lower $\tau_{75}$ of the invasive cancer samples is indicative of their metastatic capabilities.

## DISCUSSION

Currently, several independent parameters (e.g., tumor grade, stage, and subtype) are assessed histologically to help establish standard of care, but these data provide a far-from-complete assessment of tumor state and expected patient outcomes. Indeed, non-uniform distribution of genetic and phenotypic subpopulations within solid tumors causes many tumors of similar histological grade to have vastly different metastatic potential, thus complicating existing prognostic assays.^34–36^ Without more advanced detection methods, oncologists cannot provide the best recommendations for patients whose disease is marginal, low grade, or where consensus treatment options fail.^1,2^ Notably, adhesion strength has emerged as a potential biophysical marker,^22–29^ but its links to cancer metastasis have only been demonstrated *in vitro* using surrogates (e.g., migration, velocity, and persistence).^29^ Here, we showed that weak cellular adhesion correlates with increased metastatic risk in the murine *in vivo* model, demonstrating possible clinical relevance of adhesion strength as a prognostic marker. Furthermore, our dPPFC can be used as a label-free assay to accurately measure the adhesion profile in tumor and stromal biopsies, and combining histopathological assessment and adhesion strength may better correlate to clinical outcomes over time.

The dPPFC appears to have several key advantages compared to other emerging or recently FDA-approved devices. While other methods surveil CTCs in liquid biopsies via single markers to detect cancer cells that have intravasated into the blood stream,^37–41^ many have been unable to accurately predict disease severity or increase patient survival. Often, disease has already progressed, is hindered by intravasating cell heterogeneity,^42–44^ signal substantially diluted as CTCs clear quickly from the bloodstream,^45^ or cells may disseminate after assessment due to significant tumor dormancy.^9^ These considerations result in lower single marker assay specificity, sensitivity, and AUC^46^ versus stromal-based physical assays.^10,16,19,21,47,48^ While biomarker assays have also moved from single to multigene prognostic analyses (i.e., 21 to 70 genes),^49,50^ even their prediction accuracy has remained less^51^ than reported here or for other physical assays. Conversely, many physical assays have been benchmarked against large panels of normal epithelial, cancer, and transgenic cell lines that demonstrate predictive capabilities when assessing metastatic potential. Physical assays also are often label free, which can increase detection speed and throughput and reduce cytotoxicity. When distinguishing between these physical assays, the dPPFC may be faster and more robust owing to short interrogation time similar to deformation cytometry^16^ (minutes) versus migration assays within confined channels (hours to days).^10,21^ Confinement assays also require significant microfabrication and are single-use devices; dPPFC consumables are only the underlying standard-sized microscope slide. With multi-channel designs, dPPFC can still be used in moderate throughput to evaluate potential therapeutics. It also only requires 8 × 10^4^ cells to achieve an AUC of 0.94, which is an order of magnitude fewer cells than reported in other assays^10,21^ with comparable sensitivity and specificity.

Relative to other physical markers, adhesion may be a more robust *in vivo* indicator of metastatic disease. First, GFP^+^ cells that disseminated into the stroma were less adhesive, and, when cells were pre-sorted to create tumors of a single adhesive mechanotype, strongly adherent cells often resulted in very few GFP^+^ lesions in the lungs of injected NSG mice. RNA sequencing (RNA-seq) of these sorted cell types showed that cells in primary tumors up to 6 weeks *in vivo* maintained pre-injection differences,^29^ and weakly adherent cell genes associated with cell migration and locomotion ontologies that themselves were associated with shorter disease-free intervals in TCGA.^29^ Such genes could constitute the genomic signature of the adhesive mechanotype.^28,29^

Second, our dPPFC detects and quantifies cells with this adhesion mechanotype, which appears to directly correlate with metastatic tumor formation. Weakly and strongly adherent groups have different capacities to migrate within 2D and 3D assays, but not because of expression differences of focal adhesion proteins (e.g., FAK, pFAK, paxillin).^28,29^ Instead, weakly adherent cell adhesions are more dynamic and the cells themselves are more contractile than their strongly adherent counterparts with increased speed and total cell displacement. Additionally, weakly adherent cells ignore matrix stiffness gradients and migrate equally between stiff and soft environments, while the strongly adherent cell population preferentially migrates toward stiff tumor-associated matrix environments.^30^ We believe this ability to escape stiff tumor regions allows the weakly adherent cells to cause a higher number of metastatic lesions, compared to strongly adherent cells, *in vivo*. These observations also suggest that a pharmacological strategy that alters the mechanotype of a disseminating cell, perhaps via changes to its transcriptome, could render it less metastatic. Indeed, tumor cells that have acquired drug resistance have been shown to change their mechanotype, becoming more deformable and mesenchymal than their drug-sensitive counterparts.^17^

### Limitations of the study

Despite the broad implications of these findings, it is important to note ongoing limitations to dPPFC and other physical assays. First, patients often have tumors resected and then monitored for secondary disease without the primary tumor being present. Our model here primarily involved tumor progression for 6 weeks without resection; results may reflect higher-than-normal metastatic burden relative to patients receiving standard of care. Sorting cells at a variety of points in the cell cycle could also have resulted in a subset of strongly adherent cells to artificially appear weakly adherent from mitosis, biasing weakly adherent cells to appear more strongly adherent post cell division. Second, patients are typically treated with a combination of doxorubicin hydrochloride (Adriamycin) and cyclophosphamide followed by paclitaxel (Taxol), i.e., AC-T therapy,^52^ which may alter the adhesion mechanotype of disseminating cells. However, the perivascular niche can protect chemoresistant disseminating tumor cells,^53^ so, despite treatment, the adhesive mechanotype may remain intact; for example, cells can become sensitized to chemotherapy only when combined with integrin-function-blocking antibodies. Third, we assayed tumors from NSG mice, but the presence of immune cells could complicate disseminating cell signaling and dormancy; prolonged inflammation via neutrophil infiltration can awaken dormant cancer cells by cleaving extracellular matrix, activating integrins,^54^ and potentially changing adhesion mechanotype. Fourth, we measured differences in primary tumor formation within NSG mice and the number of metastatic lesions formed within the lungs, but we did not determine exactly where in the metastatic cascade weakly adherent cells outcom- pete strongly adherent cells (e.g., intravasation, circulation, extravasation). We have previously shown that weakly adherent cells are more migratory prior to injection, and this could lead to more metastatic lesions; however, differences in adhesion strength and migration could have an impact in may stages of the process. Fifth, human patient data are approaching but are not yet statistically significant. Moreover ancestry, race, and ethnicity could not be reported; hence, they could also provide variance in study data. While these concerns are important, we believe that the data shown here support further *ex vivo* assessment of human samples using the dPPFC to predict the metastatic risk to the patient. Succinctly, our results suggest that adhesion strength and cancer fraction are two label-free metrics of the device that can serve as markers of metastatic potential and be utilized in a prognostic fashion to screen patient samples.

## RESOURCE AVAILABILITY

### Lead contact

- Further information and requests for resources and reagents should be directed to and will be fulfilled by the lead contact, Adam Engler (aengler@ucsd.edu).

### Materials availability

- All unique/stable reagents generated in this study are available from the lead contact with a completed materials transfer agreement.
- Flow chambers used in this study will be made available on request as permitted.

### Data and code availability

- Bulk RNA-seq data have been deposited at GEO at GSE199785 and are publicly available as of the date of publication.
- Microscopy data reported in this paper will be shared by the lead contact upon request.
- All original code has been deposited at Zenodo at https://doi.org/10.5281/zenodo.14660022 and is publicly available as of the date of publication.
- Any additional information required to reanalyze the data reported in this paper is available from the lead contact upon request.

## STAR★METHODS

### EXPERIMENTAL MODEL AND STUDY PARTICIPANT DETAILS

#### Cell culture

Cell lines were cultured according to media conditions in Table S5 and include MDAMB-231, MDAMB-468, BT20, MCF-7, BT549, SUM1315, MCF10AT, MCF10A-DCIS, and MCF10A. All lines are female. Products were purchased from Life Technologies. Cells were obtained from ATCC or GeneCopoeia (for GFP+Luc+ MDAMB468 cells), authenticated by morphology, growth curve, karyotype, and isoenzyme analysis, and verified mycoplasma free via PCR.

#### Animal models

All animal care and experiments described below were approved by the Institutional Animal Care and Use Committee of the University of California, San Diego (study #S11102). 11-week-old female NOD/SCIDγ mice were used in this study.

#### Human subjects

Human subject protocol approval was granted by UC San Diego’s institutional review board (protocol #810860) and Moores Cancer Center’s (MCC) protocol review management committee, and MCC’s Clinical Trials Office manages the protocol as an Investigator Initiated Trial. Fresh samples of normal human breast tissue or human breast tumor were collected from 20 women of which 13 women, between 40 and 76 years of age, undergoing reduction mammoplasty, partial lumpectomy, or mastectomy at Jacobs Medical Center (San Diego, CA) were included in the study; 4 patient samples were used in assay optimization and not included in the demographic data in Table S6. Samples from 3 patients were excluded because resected tumor size was insufficient to obtain cells for the assay. Overall, 16 samples total were obtained given that some patients had bilateral mastectomies and the non-disease side was treated as a reduction sample as indicated in Table S6. Patient samples were allocated into reduction (6 samples), DCIS (5 samples), and IDC/ILC (5 samples) was determined by pathological assessment after resection. Having at least 5 samples per group was determined to be sufficient as indicated by power analysis. Additional patient demographics are reported in Table S6 but do not include ancestry, race, and ethnicity as these were deemed identifiable data by the MCC protocol review management committee. Samples were transported to lab and digested within 1 hour of receipt.

### METHOD DETAILS

#### Creating GFP and luciferase-expressing MDA-MB231 cells and validating their adhesion heterogeneity

To make lentivirus particles, HEK293T were seeded into a 20 cm dish in high glucose DMEM supplemented with 10% FBS and 1% antibiotic/antimycotic. Cells were allowed to grow until 70% confluence. At this time, 3 μg of pMD2.G (Addgene 12259), 12 μg of pCMV deltaR8.2 (Addgene 12263), and 9 mg of either GFP or luciferase plasmid (generous gift of the Kun-Liang Guan lab) was added to 1.5 mL Opti-MEM. Separately, 36 μL of Lipofectectamine 2000 was added to 1.5 mL Opti-MEM. After incubating the solutions for 15 minutes, the solutions were mixed and incubated for an additional 30 minutes. The mixture was then added dropwise to HEK293T cells. After 48 hours, media was harvested and replaced. After an additional 24 hours, media was harvested again and all media was concentrated using an Amicon Ultra-15 ultrafilter (100,000 NMWL cutoff) to a final volume of 1 mL, which was aliquoted into 250 μL aliquots and frozen at −80°C.

Media with packaged lentiviral particles was added to cultured MDA-MB231 cells along with 8 μg/mL of polybrene. After 24 hours, the media was aspirated and replaced with normal culture media. Upon observation of GFP expression through fluorescence microscopy, cultured cells were treated with 2 μg/mL of puromycin in culture media and cultured for two days. The remaining cells were sorted with a Becton Dickinson FACSAria II for presence of GFP, with unstained cells as a negative control to establish a gating strategy. All data was analyzed by FlowJo software. GFP expression was validated via fluorescence microscopy using a Nikon Eclipse Ti-S microscope at 10X magnification with FITC. To validate that adhesion heterogeneity is maintained post-transduction, weakly and strongly adherent subpopulations were isolated by exposure to pre-determined low and high shear stresses, respectively, in a microfluidic flow chamber as previously described^11^ and as detailed below. Weakly and strongly adherent cell fractions were seeded onto 2.4 mg/mL Type I collagen gels (Corning) and imaged with a Nikon Eclipse Ti-S microscope equipped with a temperature- and CO2-controlled stage for 24 hours, after which their migration was tracked and analyzed using a custom MATLAB script. Cells that divided or did not remain in the frame for 24 hours were not tracked.

#### Cell adhesion assessment

To measure population adhesion, two assays were used or developed herein: (i) the spinning disc assay, which applies shear up to 10^3^ dynes/cm^2^ as previously described,^28^ and (ii) the divergent parallel plate flow chamber (dPPFC), which enables direct visualization of detachment. To collect cells detaching at a specific shear stress, a (iii) straight walled parallel plate flow chamber (PPFC), which applies uniform shear, was developed.

- *Spinning disk assay:* GFP+ MDA-MB231 cells from tumor and stroma fractions were seeded onto 25 mm glass coverslips—coated with 2 μg/mL of fibronectin and blocked with 5% bovine serum albumin—and incubated overnight at 37°C at a cell density of 10^4^ cells/cm^2^. Cells were then exposed to shear stress at varying RPMs using the spinning disk shear assay as previously described.^28^ Cells were immediately fixed in 3.7% formaldehyde for 10 minutes, after which they were stained with 1:2000 Hoechst in DI water. Quantification of cellular adhesion strength was performed as previously described.^28^
- *Divergent parallel plate flow chamber (dPPFC)*: This device is based off the linear shear stress flow chamber from Usami and co-workers^56^ and designed using Solidworks. Chamber width was varied so that it becomes increasingly wider with chamber length so that shear stress in the device decreases exponentially, with the profile of the side walls varying according to,
(Equation 1) $$\text{w}={\text{w}}_{\min}{\text{e}}^{\left(\frac{lnln(10)}{L_{max}}L\right)}$$
  where *L* is the position along the channel, $W_{min}$ is the narrowest part of the channel, $L_{max}$ is the length between the widest and narrowest points of the channel. The wall shear stress can then be calculated at any point inside the device according to,
(Equation 2) $$\tau =\frac{6\text{Qμ}}{{\text{wh}}^{2}}$$
  Where *Q*, μ, and *h* are flow rate, viscosity, and channel height respectively. The V-shaped outlet of the channel allows the shear stress to continue to decrease to zero from where the channel is widest (Figure S7). Wall shear stress along the length of the chamber was validated by finite element analysis using COMSOL Multiphysics and agreed with values determined from Equation 2 and shown in Figure S3C. The flow chamber was fabricated out of polycarbonate (McMaster-Carr, 8707K173). The channel was made by cutting the divergent profile in 127 mm thick silicone gasket (SMI) using a craft cutter (Silhouette Cameo 4). A pocket was made from 1 μm thick adhesive backed silicone rubber (McMaster-Carr, 5787T115) to align a 25x75 mm glass slide against the gasket. A glass slide with 8x10^4^ cells (2.5x10^4^ cells/cm^2^) was then clamped down onto the gasket by a second polycarbonate plate by screwing it into the base plate.
- *Straight walled parallel plate flow chamber (PPFC) for Cell Sorting:* GFP+ Luc+ cells were seeded (~2x10^4^ cells/cm^2^) overnight onto fibronectin-coated glass slides. Using the parallel-plate flow chamber, the slides were exposed to 30 dynes/cm^2^ (for MDA-MB231 cells) or 37.5 dynes/cm^2^ (for MDA-MB468 cells) shear stress for 2 minutes to collect the weakly adherent cell fraction (weakest 25% of the population). Subsequently, the slides were exposed to 60 dyn/cm^2^ (for MDA-MB231 cells) or 75 dynes/cm^2^ (for MDA-MB468 cells) shear stress for 2 minutes to remove the middle 25% to 75% of cells. Any cells that remained on the slide (representing the most strongly adherent 25%) were removed via 0.25% trypsin and collected as the strongly adherent cell fraction. The weakly adherent and strongly adherent cell fractions were cultured separately for at least 48 hours, as well as a perfused but unsorted control population. 10^6^ GFP and Luciferase expressing MDA-MB231 cells from the strongly adherent fraction, weakly adherent fraction, or the unsorted control were suspended in 40 μL of Matrigel-PBS (1:1) mixture and were injected into the inguinal mammary fat pad of 11-week-old female NOD/SCIDγ mice. For MDA-MB468 cells, between 5x10^4^ and 10^6^ cells were injected for each of the three sorting conditions, and thus we normalized these data for injected cell density due to differences in sort parameters and yield. Tumor growth was monitored at 2-week intervals and the mice were sacrificed at 6 weeks post-injection. 6 weeks was chosen as the length of the study because few metastatic tumors were seen at 4 weeks, but the lungs were saturated with metastases at 8 weeks (Figures S5A and S5B). At time of sacrifice, the lungs and primary tumors were harvested, flash frozen or frozen in optimal cutting temperature compound, and stored at −80°C. Prior to freezing, the lungs were imaged using a GFP filter on a microscope to quantify the number of metastatic tumors.

#### Isolation of unsorted MDA-MB 231 cells in tumor and surrounding stroma

10^6^ unsorted MDA-MB 231 cells, expressing GFP and Luciferase, were suspended in 40 μL of Matrigel-PBS (1:1) mixture and were injected bilaterally into the inguinal mammary fat pads of 11-week-old female NOD/SCIDγ mice. Tumor growth was monitored at 2-week intervals and the mice were sacrificed at 6 weeks post-injection. Mice fat pads were surgically removed and dissected to separate tumor from adjacent tissue. Using an inverted fluorescent microscope, the stiffened tumor bolus was manually separated from the surrounding stroma. Both tumor and stroma were finely minced then treated with Accumax and placed on a shaker at room temperature for 2 hours. Cells were then pipetted through a 70 μm cell strainer and neutralized with culture media. For spinning disk assay cells were centrifuged and resuspended in FACS buffer (2% goat serum, 5 mM EDTA in PBS), and GFP+ cells in tumor and stroma sections were sorted via FACS.

#### RNA sequencing

Tumors were dissociated, and the resulting RNA purified using Qiagen Rneasy Mini Kit. RNA quality was quantified and assessed using TapeStation (Agilent), RNA libraries were prepared using the Illumina TruSeq Stranded RNA, High Throughput Library Prep Kit. RNA was sequenced using the Illumina HiSeq 4000 system to generate 50 bp single end reads. Data was analyzed using Rosalind, with a HyperScale architecture developed by OnRamp Bioinformatics, Inc. Reads were trimmed using cutadapt^57^ and quality scores were assessed using FastQC.^58^ Reads were aligned to the Homo Sapien genome build hg19 using STAR,^59^ while individual sample reads were quantified using Htseq and normalized via relative log expression using DESeq2 R library.^60^ DESeq2 was also used to calculate fold-changes. Clustering for the differentially expressed gene heatmap was performed via the Partitioning Around Medoids method with the fpc R library.^61^ Functional enrichment analysis of gene ontology was done using HOMER.^62^ GO terms were assigned based on PANTHER pathways.

#### The Cancer Genome Atlas dataset analysis

The Cancer Genome Atlas (TCGA) raw data were downloaded from NIH NCI GDC Data portal directly and corresponding clinical metadata were obtained from a previous publication.^55^ Only the breast cancer patients with negative histological staining for Her2, ER, and PR markers were included in our analysis cohort. Patient data was analyzed to determine correlation between gene expression corresponding to WA or SA phenotypes and 5-year survival or progression free interval (PFI). This data was analyzed by normalizing patient gene expression to z-transformed scores with respect to the differentially expressed genes within the regulation of locomotion gene ontology term (GO:0032879; Table S4) between the WA and SA subpopulations. The z-scores were summed for every patient and subsequently mapped to WA and SA categories based on mean gene expression levels. The Kaplan–Meier method was used to create survival plots comparing the 20% of individuals with the highest score to the 20% with the lowest score and significance of survival differences and PFI between groups was analyzed using the log rank test.

#### Quantification of lung metastases sizes

Images of lung metastases obtained from the GFP microscope were quantified using the ImageJ Particle Analysis plug-in. Briefly, a constant binary threshold was applied to each image to identify each incidence of a metastasis, the pixel area of each metastasis was recorded (for MDA-MB231 tumors), and the sizes were normalized to the smallest metastasis recorded. For lungs retrieved from MDA-MB468 mice, images were taken with a fluorescent microscope and the top 1% brightest pixels were used to threshold the image to count the number of metastases when particle size became insensitive, i.e., eliminating image noise, using the Particle Analysis plug-in on ImageJ. The number of metastases for each animal was determined by adding the number of identified mets on the top and bottom of each of the 5 lobes.

#### Shear threshold quantification of mammary epithelial cancer cell lines

The cell lines used to quantify shear threshold are listed in Figures 5A and 5B, consisting of a mix of mammary cancer or epithelial origin. ~4000 cells/cm^2^ were seeded onto 25x75 mm glass slides coated with 2 μg/cm^2^ of human fibronectin or rattail collagen type I and allowed to adhere overnight. Before imaging, cells were stained with Hoechst 33342 (ThermoFisher, H3570) for 10 minutes prior to imaging, and then assembled in the divergent parallel flow plate chamber. A2.1 x 45 mm region within the chamber was scanned using a Nikon Eclipse Ti-S confocal microscope at 10x in both phase and DAPI channels to count the number of cells in the chamber before shearing. The device was then connected to a syringe pump with the flow rate set to obtain a maximum shear of either 330 or 660 dynes/cm^2^. 4.5 g/L of dextrose in PBS without magnesium and calcium was used to shear cells for 3 minutes, before returning the device to the microscope to image and count the remaining cells.

The adhesion profiles for each cell line were determined by dividing the imaged region into 61 equal sized bins (775 μm long) and plotting the shear at the center of each bin versus the fraction of cells remaining after shearing. Using MATLAB’s curve fitting toolbox, the curve of the adhesion profile was then fit to these points using the following equation,

(Equation 3) $$S=e^{-{\left(\frac{\tau }{\text{λ}}\right)}^{k}}$$

where τ is the shear along the chamber, and λ and *k* are the scale and shape parameters of the Weibull distribution. The $\tau_{50},$ or the shear stress at which 50% of the cells detach can then be calculated according to,

(Equation 4) $${\text{τ}}_{50}=\lambda {\left(-lnln(0.5)\right)}^{\frac{1}{k}}$$

and the $\tau_{75},$ or the shear stress at which 75% of the cells detach can then be calculated according to,

(Equation 5) $${\text{τ}}_{75}=\lambda {\left(-lnln(0.75)\right)}^{\frac{1}{k}}$$

Co-culture experiments were performed similarly, with a 25:75, 50:50, or 75:25 mixture of MDAMB231 and MCF10A cells seeded at a total ~4000 cells/cm^2^. To predict the percentage and $\tau_{50}$ of the MDA-MB231 cells in the mixture, the adhesion profile of the combined cell types was fit to,

(Equation 6) $$S=P_{c}e^{-{\left(\frac{\tau }{\lambda }\right)}^{k}}+\left(1-P_{c}\right)e^{-{\left(\frac{\tau }{\lambda_{10A}}\right)}^{{\text{k}}_{10A}}}$$

Using the known values for $\lambda_{10A}$ and $k_{10A}$ from the adhesion profile MCF10A cell line, the fraction of MDAMB231 cells, $P_{c}$, and their λ and *k* can be predicted from the curve fit.

#### Cell speed and displacement measurements

Cell speed and displacement was measured using timelapse microscopy on cells migrating on 3.2 kPa polyacrylamide gels. The Young’s modulus of the gel was validated using atomic force microscopy. Prior to seeding cells, gel surfaces were functionalized with collagen I (150 μg/ml) using sulfosuccinimidyl 6-(4’-azido-2′-nitrophenylamino)hexanoate (0.2 mg/ml, Sulfo-SANPAH; Pierce) as a crosslinker to the PA, and allowed to incubate at 37μC overnight. Cells were seeded at ~1500 cells/well and allowed to adhere to the PA gels overnight. The cells were then imaged for 15 hours using a Nikon Eclipse Ti-S microscope equipped with a temperature and CO2 controller (Pathology Devices Inc., LiveCell). Images were taken every 5 minutes using phase contrast at 10x. The cell trajectories were then traced using a custom MATLAB script, and dividing cells were excluded in the analysis. Cell speed was calculated by dividing the path length by the 15-hour runtime and displacement by finding the distance between the starting and ending position of the cell.

#### Dissociation and adhesion quantification of tumor, stroma, and mammary fat pad

Tumor, stroma, and the tissue from the contralateral fat pad were resected from mice sacrificed at 6 weeks post GFP+ Luc+ MDAMB231 cell injection. The tumor and stroma were separated as described for FACS sorting. All tissues were finely minced and the cells were dissociated in a collagenase solution comprised of 2mg/ml trypsin, 2mg/ml collagenase, 5% FBS, 50μg/ml gentamicin, 5μg/ml insulin, 5% fetal bovine serum, and DMEM/F12. GFP+ cells from the tumor were not FACS sorted to better recapitulate tumor samples obtained from a patient biopsy, and to test the flow chamber’s ability to distinguish between cancer and healthy cells. All of the dissociated mouse and cancer cells were then seeded and allowed to grow in a 6 well plate, for 2-3 days or until there were enough for seeding onto 25x75mm fibronectin coated glass slides. The cells were then sheared in the flow chamber and the adhesion profile for each sample was determined as described above. Tumor and stroma adhesion profiles were fit to Equation 6 to predict the $\tau_{50}$ and percentage of cancer cells in these samples, using the λ and *k* values measured from the adhesion profile of the contralateral fat pad in place of those from the MCF10A cells.

#### Logistic regression model and receiver operating characteristic (ROC) analysis

Metastatic risk was defined as either high or low by a threshold number of GFP+ lung nodules. For the stroma samples, using a threshold of 2 nodules, and comparing the $\tau_{50}$ and cancer fractions of mice with nodule counts above and below that threshold, gave us the most significant difference by a Wilcoxon rank sum test between the two groups. The binary response variable was then defined as low risk for mice with ≤1 GFP+ lung nodules and high risk for mice with >1 GFP+ lung nodule. A logistic linear regression model was then fit to obtain the probability estimates of having more than 1 tumor based on the following equation,

(Equation 7) $$p=logit(y)=a+b\tau_{50}+cP_{c}+d\tau_{50}P_{c}$$

where *a*, *b*, *c*, and *d* are the estimated coefficients from MATLAB’s generalized linear regression model, and $\tau_{50}$ and $p_{c}$ are the median detachment shear and cancer fraction respectively. A deviance test was used to compare whether the model differs significantly from a constant model. Finally, parameterized data was used to create a Receiver Operator Characteristic (ROC) curve, which scores the diagnostic ability of binary classifiers. Here, we used probability estimates from the logistic regression model as the classifier scores to obtain the ROC curve and area under the curve (AUC) using MATLAB’s perfcurve function. The True Positive Rate (y-axis) indicates the sensitivity of the assay to correctly assess an outcome based on the ground truth (i.e., the binary classifiers), the False Positive Rate (x-axis) indicates the specificity of the assay such that it does not incorrectly classify negative outcomes, and the AUC indicates the probability that a binary classifier will rank a random positive sample higher than a random negative sample; AUC values close to 1 indicate that an assays could be a strong diagnostic tool.

#### Dissociation and preparation of cell suspensions from tissue samples

To obtain single cell suspensions, tumor or normal breast tissue were treated according to a digestion method previously described.^63^ Briefly, the tissue was rinsed with PBS and minced into a pulp. Following mechanical digestion, digestion medium (DMEM/F12 (1:1) supplemented with 1 mg/mL BSA, 2 mM glutamine, 100 U/mL Penicillin/Streptomycin, 300 U/mL collagenase I, 100 U/mL hyaluronidase, 10 ng/mL epidermal growth factor (EGF), 100 ng/mL cholera toxin, 5 ug/mL insulin, 0.5 ug/mL hydrocortisone, and 5% Fetal Bovine Serum (FBS)) was added to pulp and transferred to a 15 mL conical to rotate for 18 hours at 37°C. After, the tissue was centrifuged at 700 g for 5 minutes and the supernatant containing the adipose cells was discarded. The remaining pellet was resuspended in ACK lysis buffer to lyse red blood cells (RBC). Following RBC lysis, the suspension was centrifuged at 700g for 5 minutes, supernatant was discarded post centrifugation and 0.25% Trypsin-EDTA was added to pellet to further break tissue into a single cell suspension. Trypsin-EDTA was deactivated after a 3-minute incubation and suspension was centrifuged at 700g for 5 minutes. Post centrifugation the supernatant was removed, and the pellet was resuspended in a dispase-DNase I solution (5 U/mL and 100 ug/mL respectively) and incubated for 3 minutes. After the incubation, the suspension was centrifuged at 700g for 5 minutes, supernatant was removed, and the pellet was resuspended in growth media (DMEM/F-12 + 10% FBS + 0.5 ug/mL hydrocortisone, 20 ng/mL hEGF, 10 ug/mL Insulin, 100 ng/mL cholera toxin + 1% Penicillin/Streptomycin). The cell suspension was subsequently filtered through a 70-um cell strainer and cells were seeded into tissue culture treated flasks. Cells were grown for one passage prior to being used for shear assays.

#### Shear threshold quantification of primary cells from human tissue

The methodology used to shear the primary cells is the same as previously mentioned in section shear threshold quantification of mammary epithelial cancer cell lines. Briefly, 80,000 cells/cm^2^, on passage 2, were seeded onto 25x75 mm glass slides coated with 2 ㊼g/cm^2^ of human fibronectin and allowed to adhere overnight. The following day cells were stained with Hoechst 33342 for 10 minutes and then assembled in the divergent parallel flow plate chamber. Pre shear images of the device were taken to observe the number of adherent cells prior to flow exposure. Subsequently the device was connected to a syringe pump with the flow rate set to obtain a maximum shear of 660 dyn/cm^2^. 4.5 g/L of dextrose in PBS without magnesium and calcium was used to shear cells for 3 minutes, before returning the device to the microscope to image and count the remaining cells. The adhesion profiles for the human cells were determined using the pre- and post-shear images and fitting these points using Equation 3. Technical replicates which had an R^2^>0.5 were averaged together to obtain an averaged adhesion curve. The $\tau_{75}$, or the shear stress at which 50% of the cells detach, can then be calculated according to Equation 4. The standard error of the mean was calculated for each patient’s averaged adhesion curve and plotted. Additionally, the upper and lower 95% prediction and confidence bounds for the averaged adhesion curve were calculated using Matlab’s predint and confint functions. Prediction bounds were plotted on averaged adhesion curve and the confidence interval for the $\tau_{75}$ was reported.

#### Immunostaining of primary cells from human tissue

Cells were harvested at passage 2 and seeded on 12 mm coverslips at a density of 8400 cells/cm^2^. 24 hours post seeding cells were fixed with 4% paraformaldehyde for 15 minutes at room temperature. The samples were then washed 3 times with PBS and then blocked with 2% BSA for 1 hour at room temperature. After blocking cells were incubated with rabbit anti-E-cadherin (Cell Signaling, 24E10; 1:200) or rabbit anti-PPARγ (Proteintech, 16643-1-AP; 1:200) and mouse anti-CD31 (Zeta Corporation, Z2136MS; 1:50) or mouse anti-TE7 (1:100) or mouse anti-EPCAM (Invitrogen, 14-9326-82; 1:100) overnight at 4C. The cells were then washed 3 times with PBS and then incubated with donkey anti-rabbit 488 (Life Technologies, A21206; 1:500) and donkey anti-mouse 647 (Life Technologies, A31571; 1:500) for two hours at room temperature. Following incubation with secondary antibody, cells were washed once with PBS and then incubated with Hoechst 33342 (ThermoFisher, H3570; 1:1000) and rhodamine phalloidin (Biotium, 00027; 1:500) for 30 minutes at room temperature. Cells were washed 3 times with PBS and then mounted using Fluoromount-G (SouthernBiotech, 0100-01) on microscope slides. Images were taken using a Keyence Fluorescence Microscope Bz-X series using a 20× objective.

### QUANTIFICATION AND STATISTICAL ANALYSIS

For all analyses, †*p* < 0.1; *p < 0.05; **p < 0.01; ***p < 0.001; and ****p < 0.0001. Figures 1B–1D, 3C, and S1C were performed using two-tailed unpaired t test. Figures 2B, 3B, and 7G was analyzed using a one-way ANOVA with Tukey test for multiple comparisons. Data expressed as box-and-whisker plots show all points with the whisker ends corresponding to minimum and maximum values. All other values are expressed as mean ± SD. Statistical analyses were performed using GraphPad Prism Software v9.0.

## Supplementary Material

1

2

3

## Figures and Tables

**Table:** KEY RESOURCES TABLE

REAGENT or RESOURCE	SOURCE	IDENTIFIER
Antibodies		
Rabbit monoclonal Anti-E-Cadherin	Cell Signaling	Cat#3195; RRID: AB_2291471
Rabbit polyclonal Anti-PPAR Gamma	Proteintech	Cat#16643-1-AP; RRID: AB_10596794
Mouse monoclonal Anti-CD31	Zeta	Cat#Z2136MS
Mouse monoclonal Anti-Fibroblasts	Millipore Sigma	Cat#CBL271; RRID: AB_93449
Mouse monoclonal Anti-CD326 (EpCAM)	Invitrogen	Cat#14-9326-82; RRID: AB_795876
Donkey polyclonal Anti-Rabbit	Invitrogen	Cat#A-21206; RRID:AB_2535792
Donkey polyclonal Anti-Mouse	Invitrogen	Cat#A-31571; RRID: AB_162542
Bacterial and virus strains		
pMD2.G	Addgene	RRID:Addgene_12259
pCMV delta R8.2	Addgene	RRID:Addgene_12263
GFP	Generous gift of the Kun-Liang Guan lab	N/A
Luciferase	Generous gift of the Kun-Liang Guan lab	N/A
Biological samples		
Healthy adult breast tissue	Jacobs Medical Center at UC San Diego Health	N/A
Ductal carcinoma *in situ* patient specimens	Jacobs Medical Center at UC San Diego Health	N/A
Invasive ductal/lobular carcinoma patient specimens	Jacobs Medical Center at UC San Diego Health	N/A
Chemicals, peptides, and recombinant proteins		
Hoescht 33342	Invitrogen	Cat#H3570
Rhodamine Phalloidin	Biotium	Cat#00027
Cholera Toxin	List Biologicals	Cat#100B; CAS: 9012-63-9
Hyaluronidase	Sigma	Cat#H3506; CAS: 37326-33-3
Hydrocortisone	Sigma	Cat#H4001; CAS:50-23-7
Insulin	Lonza	Cat#BE02-033E20
Dispase II	Gibco	Cat#17105-041; CAS:9001-92-7
DNase I	Worthington Biochemical Corporation	Cat#LS006333 ; CAS: 9003-98-9
Human Epidermal Growth Factor	Biolegend	Cat#585506;
ACK Lysing Buffer	Quality Biological	Cat#118-156-101
DMEM/F12	Gibco	Cat#11320033
DMEM	Gibco	Cat#11965092
Penicillin-Streptomycin	Gibco	Cat#15140122
0.25% Trypsin	Gibco	Cat#25200-056
Horse Serum	Omega	Cat#DH-05
Fetal Bovine Serum	Omega	CAT#FB-01
0.05% Trypsin	Gibco	Cat#25300-054
L-Glutamine	Gibco	Cat#25030081
Dextrose Anhydrous	Fisher Bioreagents	Cat#BP350-1; CAS: 50-99-7
Bovine Serum Albumin	Goldbio	Cat#A-420-500; CAS: 9048-46-8
Accutase	Innovative Cell Technologies	Cat#AT-104
Accumax	Innovative Cell Technologies	Cat#AM-105
Rat Tail Collagen I	Enzo	Cat#ALX-522-435-0100
Acrylamide (40%)	Thermo Scientific	Cat#J62480-AP; CAS: 79-06-1
bis-Acrylamide	Research Products International	Cat# A11275-500, CAS: 110-26-9
Ammonium Persulfate	Fisher Bioreagents	Cat#BP179-100; CAS: 7727-54-0
TEMED	Fisher Scientific	Cat#BP150-20; CAS:110-18-9
Sulfo SANPAH Crosslinker	CovaChem	Cat#13414-100
Trypsin Powder	Worthington Biochemical Corporation	Cat#LS003707
Gentamycin Sulfate USP Grade	Avantor	Cat# 97061-370
Rat Collagen I	Corning	Cat#354236
Collagenase Type I	Worthington Biochemical Corporation	Cat#LS004196
Critical commercial assays		
RNeasy Mini Kit	Qiagen	Cat#74104
Deposited data		
RNA sequencing data	This paper	GEO: GSE199785
Cancer Genome Atlas (TCGA) raw data	NIH NCI GDC Data portal	N/A
Clinical Metadata	Liu et al.^55^	N/A
Experimental models: Cell lines		
MDA-MB231	ATCC	ATCC HTB-26
MDA-MB468	ATCC	ATCC HTB-132
GFP+LUC+MDA-MB468	GeneCopoeia	Cat#SL027
MCF7	ATCC	ATCC HTB-22
MCF10A	ATCC	ATCC CRL-10317
MCF10A-DCIS	Generous gift from Dr. Jing Yang	N/A
MCF10AT	Generous gift from Dr. Jing Yang	N/A
BT549	ATCC	ATCC HTB-122
SUM1315	Generous gift from Dr. Thea Tlsty at UCSF	N/A
BT20	ATCC	ATCC HTB-19
Experimental models: Organisms/strains		
Mouse: NOD.Cg-*Prkdc^scid^Il2rg^tm1Wjl^*/Sz	UC San Diego ACP and Jackson Labs	Cat#005557; RRID:IMSR_JAX:005557
Software and algorithms		
ImageJ		https://imagej.nih.gov/ij/
Logistic Regression and ROC Analysis Script	Matlab, This Paper	https://github.com/MadisonKane-EnglerLab/Adhesion/releases/tag/v1.1
Mouse and Human Divergent Parallel Plate Flow Chamber Analysis	Matlab, This Paper	https://github.com/MadisonKane-EnglerLab/Adhesion/releases/tag/v1.1
Migration Analysis Versus T50 Data	Matlab, This Paper	https://github.com/MadisonKane-EnglerLab/Adhesion/releases/tag/v1.1
Rosalind		N/A

## Highlights

- Weakly adherent cells are the primary drivers of metastases in a murine model
- A divergent parallel-plate flow chamber can provide label-free adhesive signatures
- Adhesion retrospectively predicts metastatic disease within an *in vivo* murine model
- Adhesion strength stratifies patient samples into disease and non-disease subtypes
