# Supplementary material for: Adhesion strength of tumor cells predicts metastatic disease in vivo
Source: Cell Rep. Author manuscript; Available in PMC 2025 Apr 23. (PMC12014391; doi:10.1016/j.celrep.2025.115359)
Supplement: 1 [file NIHMS2069110-supplement-1.pdf]

Cell Reports, Volume *44*

Supplemental information

Adhesion strength of tumor cells predicts metastatic disease *in vivo*

Madison A. Kane, Katherine G. Birmingham, Benjamin Yeoman, Neal Patel, Hayley Sperinde, Thomas G. Molley, Pranjali Beri, Jeremy Tuler, Aditya Kumar, Sarah Klein, Somaye Zare, Anne Wallace, Parag Katira, and Adam J. Engler

Supplemental Data Figures

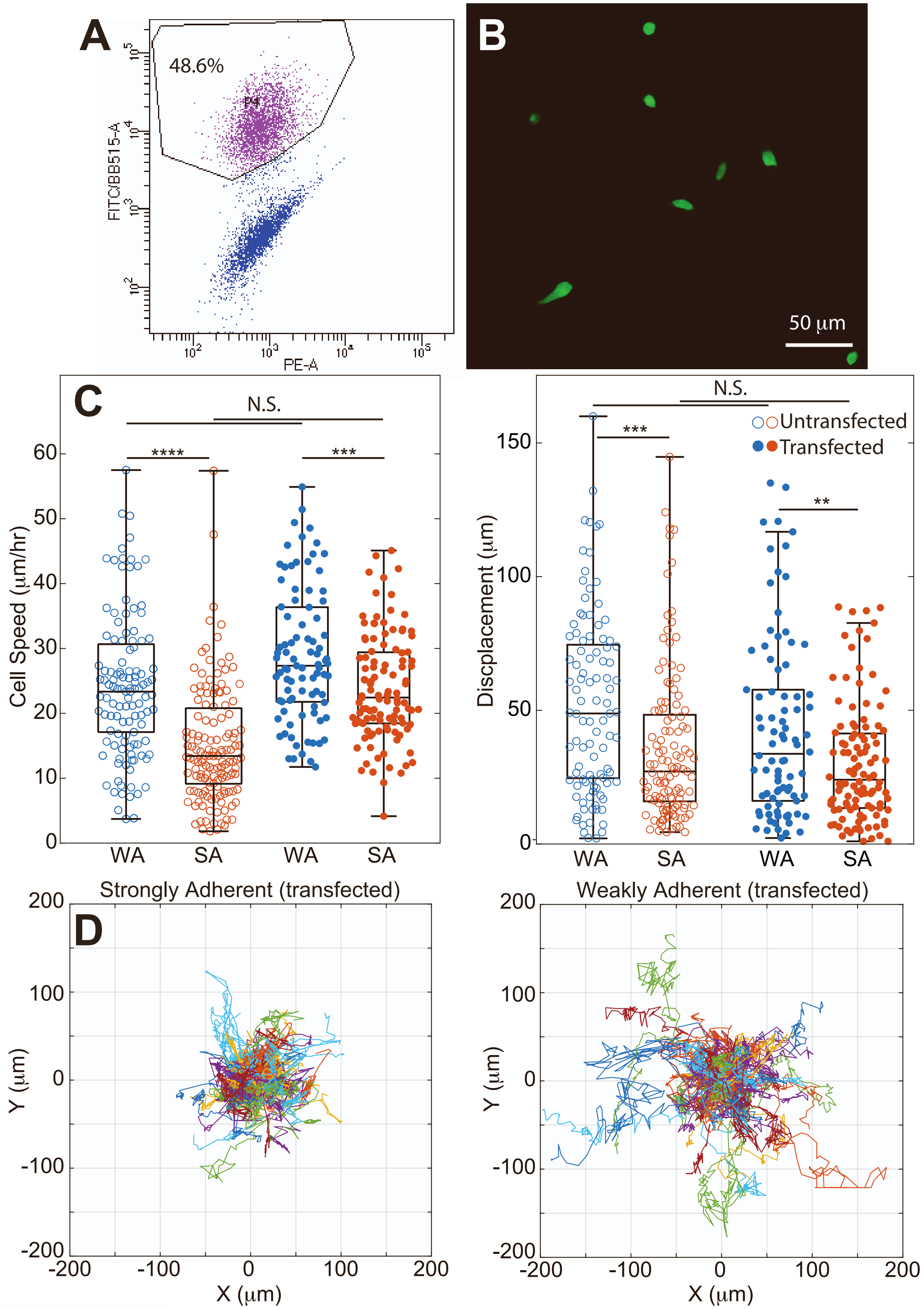

Supplemental Data Figure 1: GFP-Luciferase lentiviral transduction does not alter inherent heterogeneity of MDA-MB231 cells, Related to Figure 1. (A) After treatment with puromycin

to select for cells that expressed Luciferase, cells were sorted using FACS for GFP+ signal (y-axis). Gating strategy is based on excluding cells from an unlabeled control sample. (B) GFP expression was verified using fluorescence microscopy. Scale bar is 50 µm. (C) GFP+ weakly adherent cells were more migratory than their strongly adherent counterparts (solid data points) and similar to untransfected cells (open data points). Statistical analysis via unpaired t-test. **represents $p<0.01$. ***represents $p<0.001$ (n=89 and 111 for GFP+ weakly and strongly adherent, respectively; n=120 and 141 for untransfected weakly and strongly adherent). (D) Rose plots of GFP+ weakly and strongly adherent cells as indicated. In panel C, box and whisker plots show boxes indicating the 25th to 75th percentiles, mean as a line within the box, and whiskers indicating the minimum and maximum spread of the data.

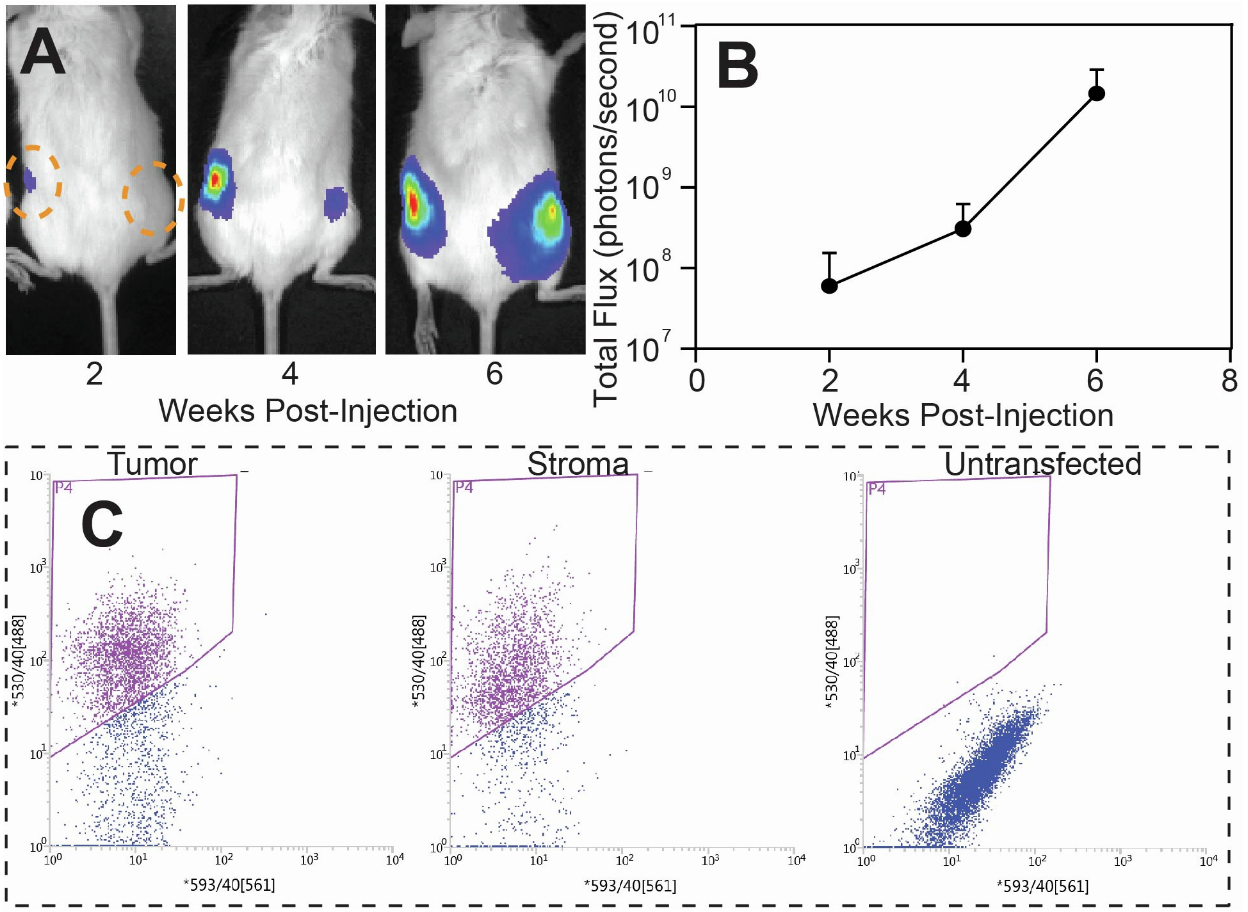

Supplemental Data Figure 2: Tumor growth was monitored using IVIS and GFP+ cells can be sorted from tumor and stroma, Related to Figure 1. (A) Tumor growth was monitored using IVIS at 2-week intervals using (B) total flux as a measurement (n=6 mice). (C) After manual separation of the stiff tumor from the surrounding stroma and dissociation into single cells, GFP+ cells (y-axis) could be isolated from both tissue fractions using FACS. Error bars in panel B are expressed as mean ± standard deviation.

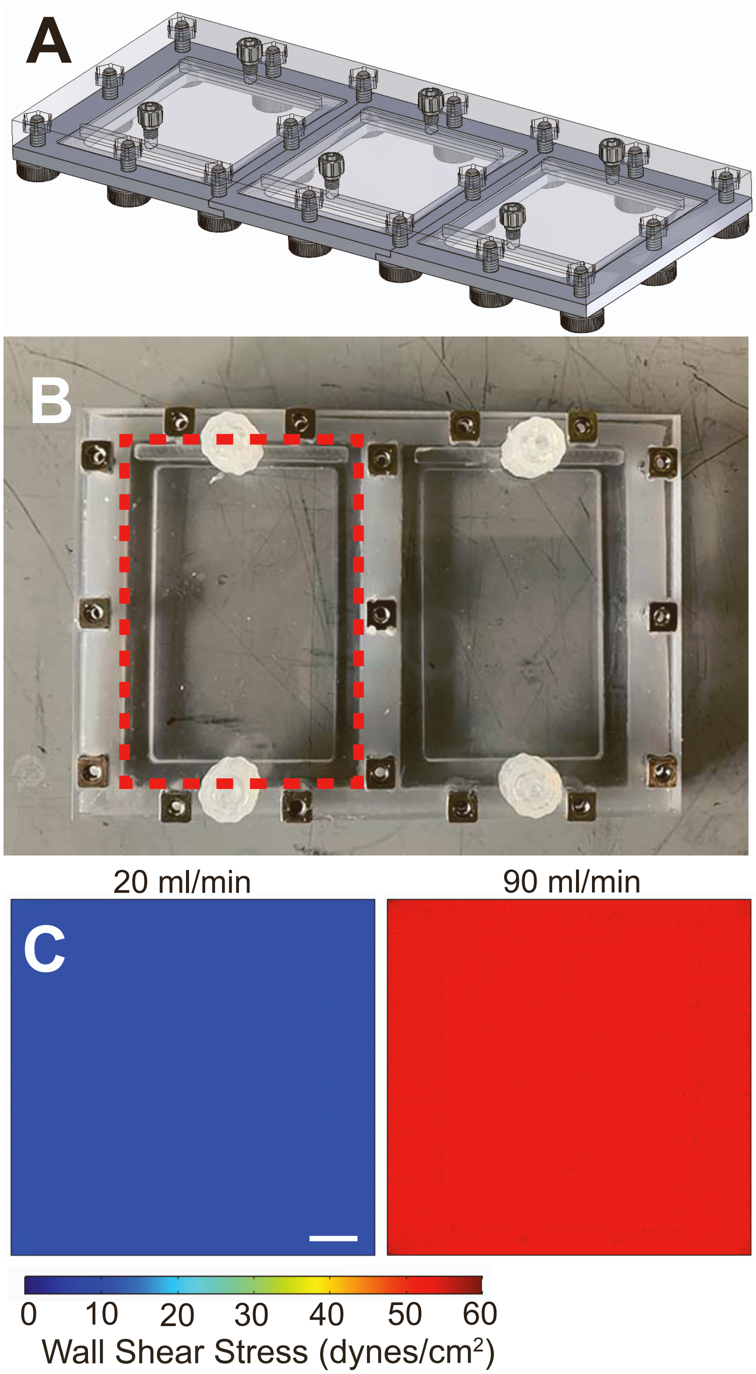

Supplemental Data Figure 3: Parallel plate flow chamber applies a uniform shear stress to cells seeded in the device, Related to Figure 2. (A) SolidWorks design and (B) top-down view of a straight walled parallel plate flow chamber (PPFC). (C) COMSOL simulation of the shear stress profile through the straight-walled parallel plate flow chamber (PPFC). The dashed line in panel B indicates the region simulated in panel C.

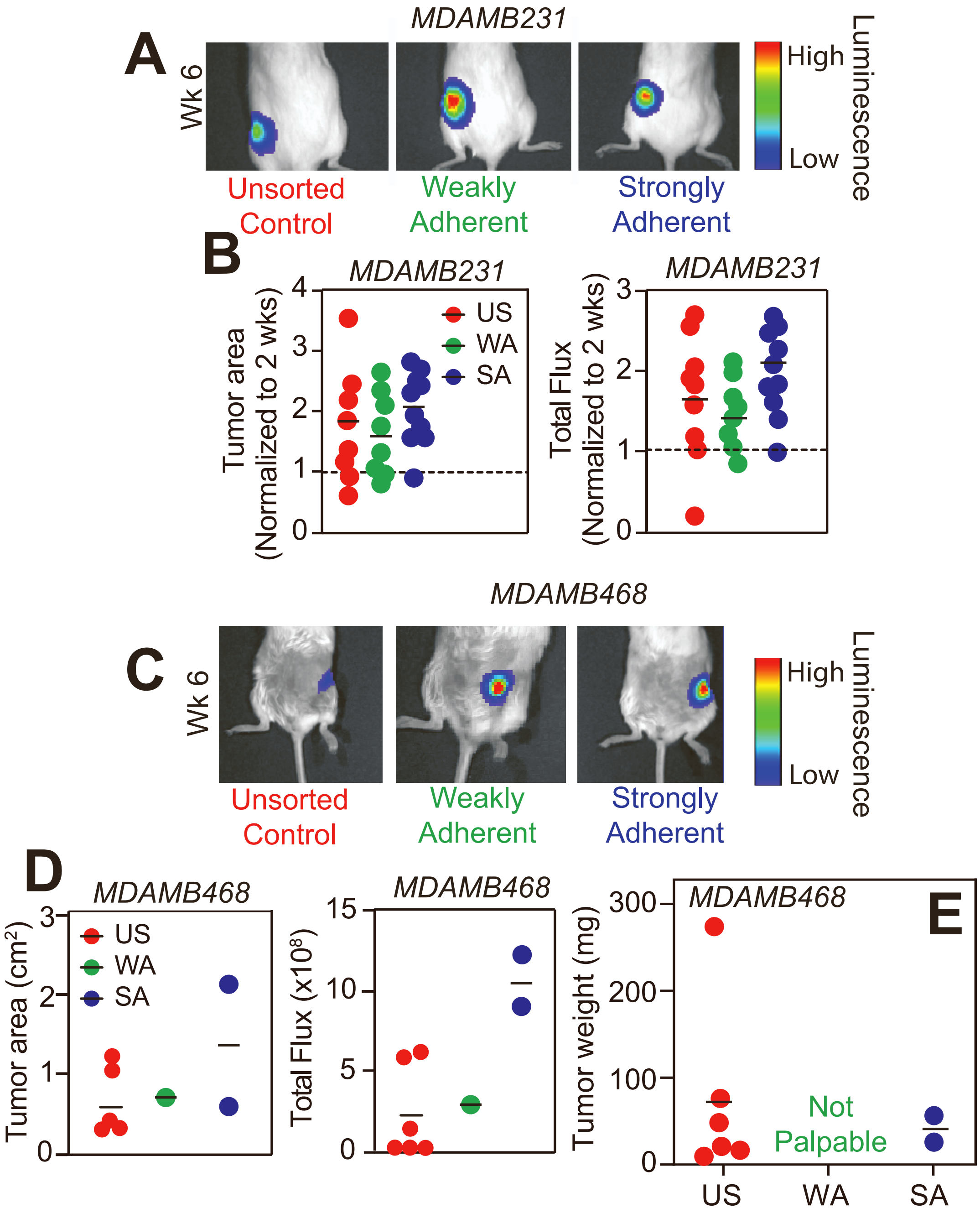

Supplemental Data Figure 4: Tumor growth in NSF mice from each adhesion type, Related to Figure 2. (A) Representative IVIS imaging of mice injected with unsorted (US), weakly adherent (WA), or strongly adherent (SA) MDA-MB231 cells for left, middle, and right columns, respectively, at week 6. (B) Quantification of the IVIS signal at week 6 normalized to week 2 data for both tumor area and total flux for mice receiving the indicated MDA-MB231 cells. Dashed line indicates no change over time. n=9, 8, and 10 for unsorted, weakly adherent, and strongly adherent cell injections, respectively. (C) Representative IVIS imaging of mice injected with unsorted (US), weakly adherent (WA), or strongly adherent (SA) MDA-MB468 cells for left, middle, and right columns, respectively, at week 6. (D) Quantification of the IVIS signal at week 6 for both tumor area and total flux for mice receiving the indicated MDA-MB468 cells. (E) Tumor weight at time

of sacrifice 6-weeks post injection for mice receiving MDA-MB468 cells. For panels D and E, n=8, 3, and 3 for mice with unsorted, weakly adherent, and strongly adherent cells injected, respectively, but only tumors with measurable data are shown, e.g., weakly adherent MDA-MB468 cells produced no palpable tumors at 6 weeks so no weight measurement was made. Statistical analysis via one-way ANOVA with Tukey test for multiple comparisons did not show significance. Lines in panel B, D, and E represent mean value of the distributions.

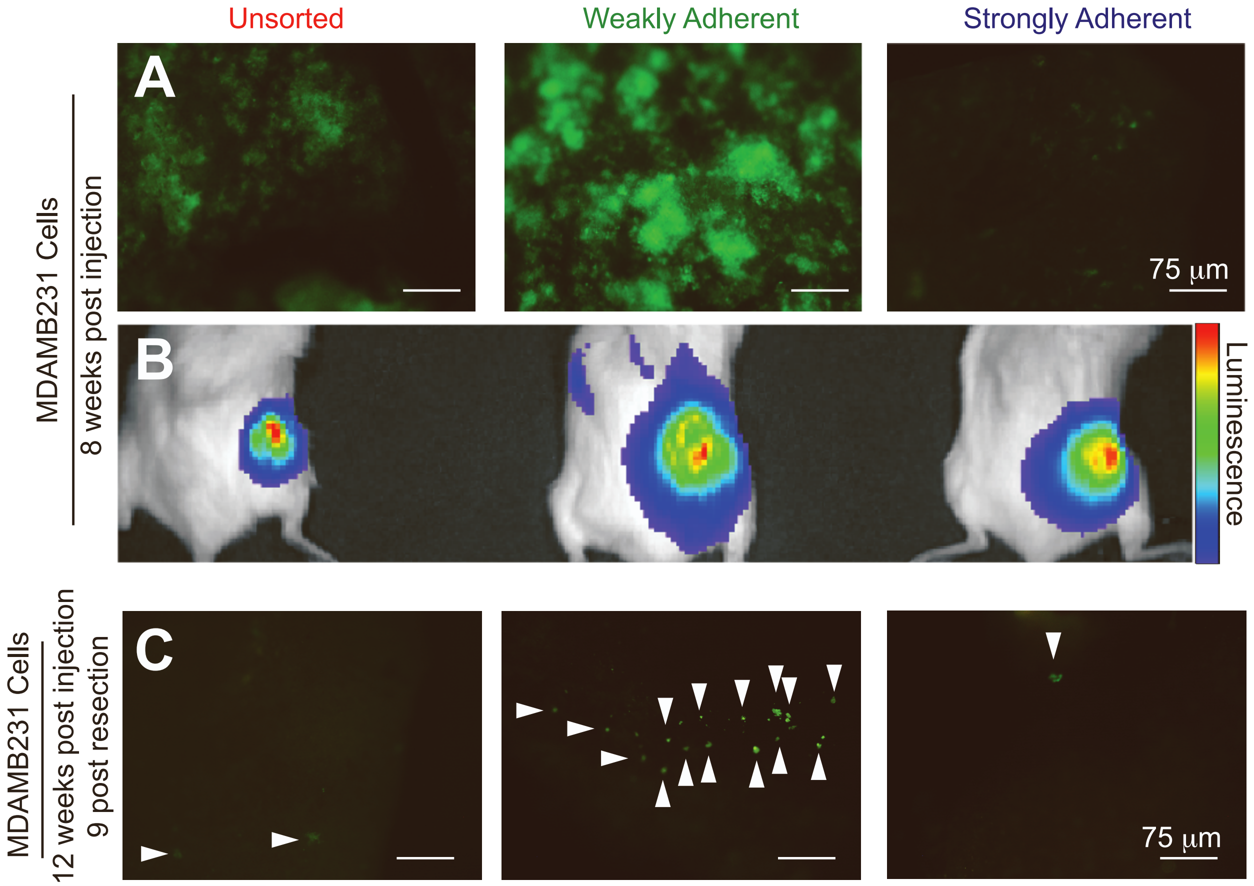

Supplemental Data Figure 5: Assessment of lung metastases between 8- and 12-weeks post-injection, Related to Figure 4. (A) Representative images of GFP+ MDA-MB231 cell-based lung metastases in mice sacrificed 8 weeks post-injection. Scale bar is 75 µm. (B) Representative IVIS image of mice 8 weeks post-injection. (C) Representative images of GFP+ MDAMB231 cell-based lung metastases in mice sacrificed at 12 weeks, which occurred 9 weeks post resection. Arrowheads indicate metastases. Scale bar is 75 µm.

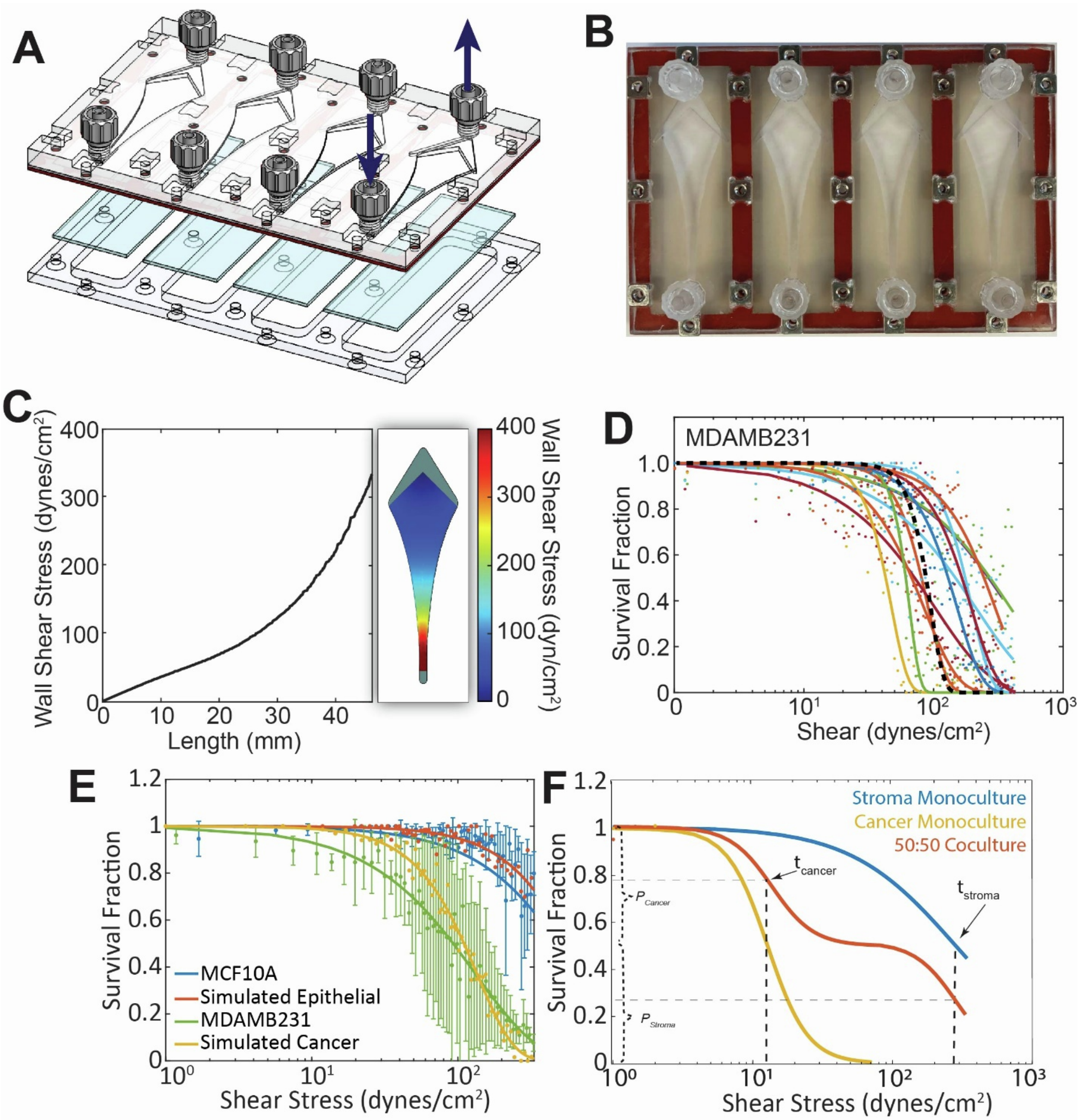

Supplemental Data Figure 6: Divergent parallel plate flow chamber applies an increasing shear stress to cells seeded in the device, Related to Figure 5. (A) SolidWorks design, (B) top-down view, and (C) COMSOL simulation of the shear stress profile through the imaging, divergent parallel plate flow chamber (dPPFC). (D) Percent of attached cells versus shear stress value in the imaging dPPFC for various replicates of perfusion of MDA-MB231 cells. Black dashed line represents the average shear stress plot of the replicates (n=12 replicates; >500 cells/replicate). (E) Comparison of statistically generated adhesion profiles and experimental adhesion profiles (n=3 replicates/cell line). (F) Schematic of adhesion profile to highlight how cancer fraction, P_c, and adhesion strength are plotted on an adhesion metric with the dPPFC; dashed lines indicate P_{cancer} and P_{Stroma} and darker dashed lines represent median cell adhesion strength for a given population. Error bars in panel E are expressed as standard deviation.

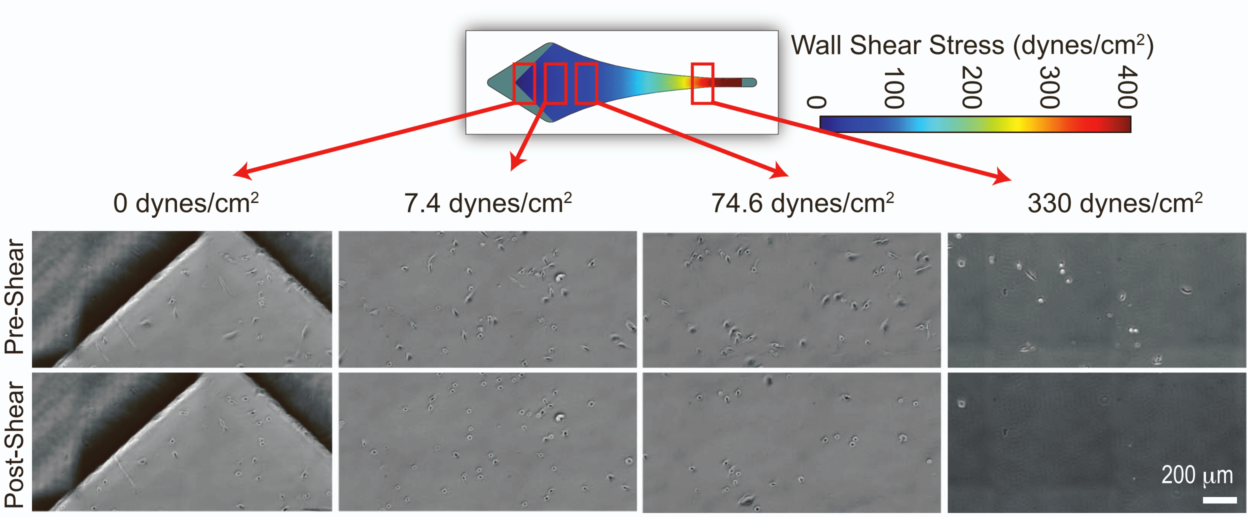

Supplemental Data Figure 7: Images of Cells in the Divergent parallel plate flow chamber, Related to Figure 5. Images of cells adhered to the dPPFC before shear exposure (top) and after (bottom) ordered from left to right in terms of increasing shear stress within the device. Scale bar is 200 µm.

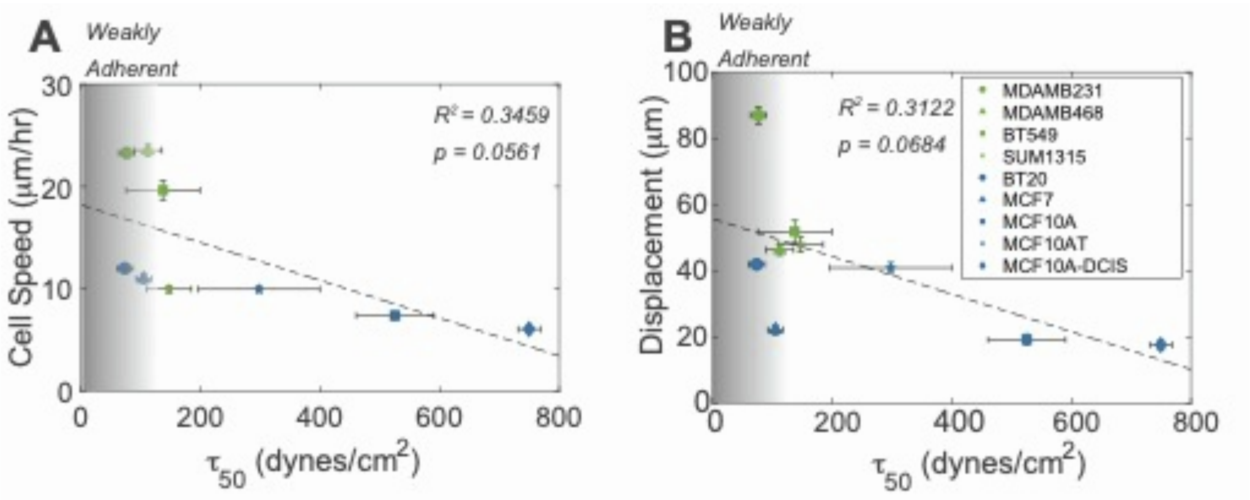

Supplemental Data Figure 8: Relationship of cell speed and displacement to adhesion strength to Collagen Type I across multiple metastatic and non-metastatic lines, Related to Figure 5. (A) Cell speed and (B) displacement on collagen gels versus shear stress, i.e., τ_{50}, when attached to collagen-coated dPPFC chambers was plotted for various metastatic (green) and non-metastatic cell lines (blue). For cell speed and displacement, metastatic cell lines (green) had n = 553, 475, 137 and 306 cells analyzed for MDA-MB231, MDA-MB468, BT459, and SUM1315, respectively. For cell speed and displacement on collagen gels, non-metastatic cell lines (blue) had n = 609, 253, 253, 225, and 305 cells analyzed for BT20, MCF7, MCF10A, MCF10AT, and MCF10AT-DCIS, respectively. For adhesion strength, metastatic cell lines (green) had n = 8, 7, 7, and 11 replicates for MDA-MB231, MDA-MB468, BT459, and SUM1315, respectively. For adhesion strength, non-metastatic cell lines (blue) had 12, 8, 5, 4, and 4 replicates for BT20, MCF7, MCF10A, MCF10AT, and MCF10AT-DCIS, respectively. All metastatic and non-metastatic cell lines have adhesion curves with a $R^2 \geq 0.5$, except MCF10A-DCIS where not all curves had $R^2 \geq 0.5$. Y-axis error bars denote the standard deviation and x-axis error bars denote the standard error of the mean.

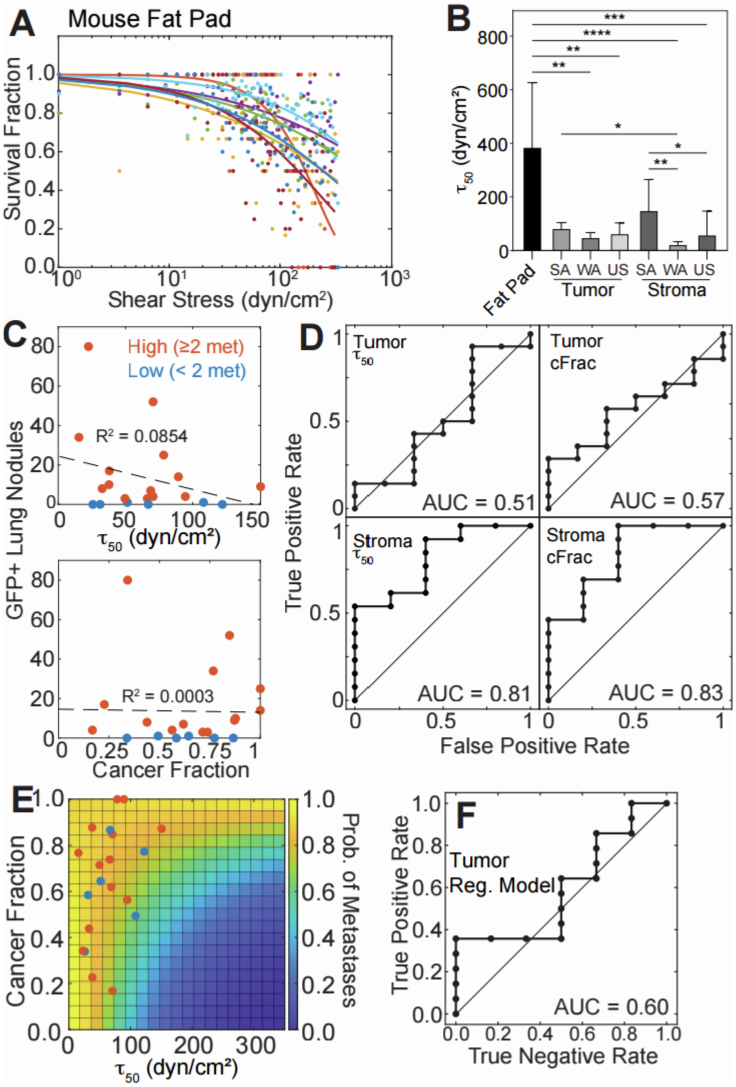

Supplemental Data Figure 9: Raw data from mammary epithelial cancer adhesion assays, Related to Figure 6. (A) Shear plots of replicates of cells dissociated from the contralateral mammary fat pad (n=7 mice). (B) Average shear stress of cells dissociated from the contralateral mammary fat pads, tumors, and surrounding stroma (n=7 mice/condition). (C) Average shear stress and cancer fraction vs. GFP+ lung nodules from resected tumor samples (n=20 lungs). (D) ROC curves of metastatic risk predictions for average shear stress or cancer fraction for resected tumor and stroma samples. (E) Logistic regression model showing probability estimate of a mouse having ≥2 tumor based on the average shear stress and cancer fraction for tumor sample. (F) ROC curve of metastatic risk predictions based on model's probability estimates. Red points are classified as high metastatic risk, i.e., ³2 GFP+ nodules, and blue are low, i.e., <2 GFP+ nodules. Bar graphs and error bars in panel B are expressed as mean ± standard deviation.

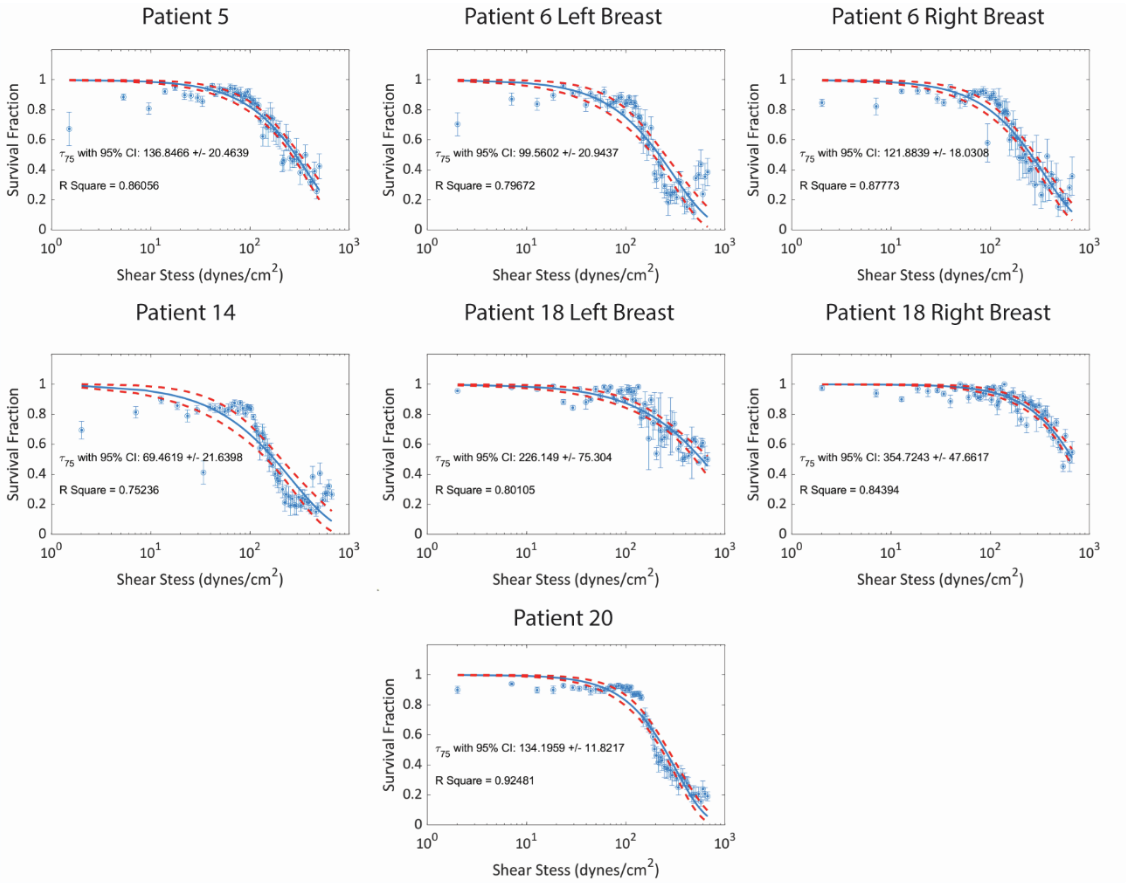

Supplemental Data Figure 10: Error plots of averaged patient adhesion curves for reduction mammoplasty samples, Related to Figure 7. Technical replicates for each patient adhesion run were averaged together to generate average adhesion curves shown in blue and 95% confidence intervals shown in dashed, dark red lines. Numerical values for τ_{75} with 95% confidence intervals are shown on the left for fits of averaged technical replicates with R^2 of the fit also shown. Patient demographics and details are shown in Supplemental Data Table 6. Data and error bars for individual data points are expressed as mean ± standard error of the mean.

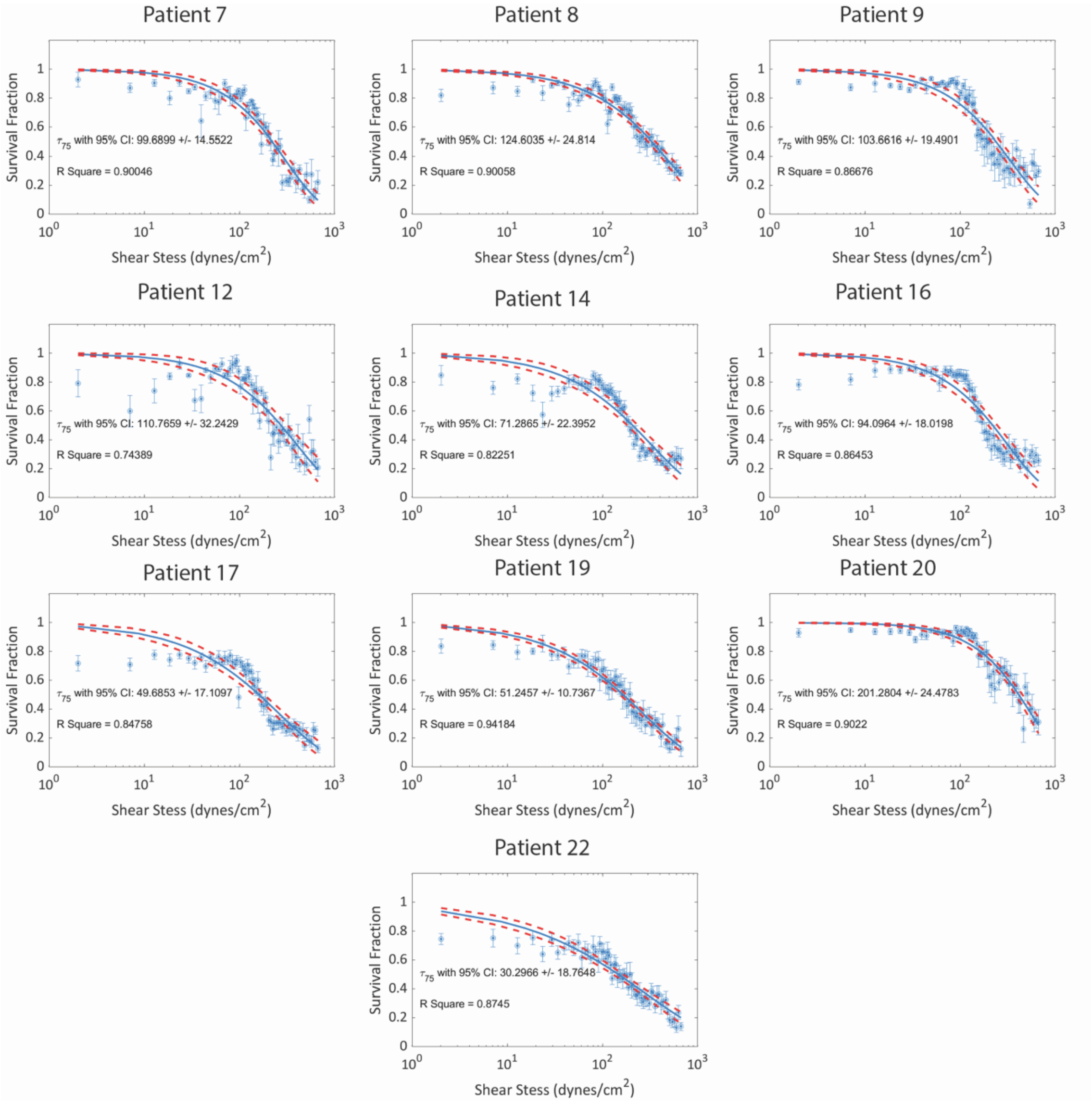

Supplemental Data Figure 11: Error plots of averaged patient adhesion curves for human tumor samples, Related to Figure 7. Technical replicates for each patient tumor adhesion run were averaged together to generate average adhesion curves shown in blue and 95% confidence intervals shown in dashed, dark red lines. Numerical values for τ_{75} with 95% confidence intervals are shown at left for fits of averaged technical replicates with R^2 of the fit also shown for tumor samples. Patient demographics and details are shown in Supplemental Data Table 6. Data and error bars for individual data points are expressed as mean ± standard error of the mean.

Supplemental Tables, Titles, and Legends

Supplemental Data Table 3: Intersection of migration and locomotion GO terms between primary tumors and pre-sorted cells, Related to Figure 2. Top GO terms (as assigned by Panther) between in vitro and in vivo primary tumors and their corresponding p-Values as plotted in Figure 2E. Data is ordered by in vivo p-value.

GO Term	In Vivo GO p-Val	In Vitro GO p-Val
anatomical structure morphogenesis (GO:0009653)	1.30980392	12.73992861
animal organ development (GO:0048513)	1.343901798	11.19722627
anatomical structure formation involved in morphogenesis (GO:0048646)	1.366531544	3.93930216
tube development (GO:0035295)	1.370590401	1.440093375
system development (GO:0048731)	1.37161107	3.358525889
regulation of multicellular organismal process (GO:0051239)	1.373659633	2.943095149
regulation of localization (GO:0032879)	1.385102784	1.609064893
regulation of cellular component movement (GO:0051270)	1.554395797	10.38299966
tube morphogenesis (GO:0035239)	1.879426069	10.48148606
multicellular organism development (GO:0007275)	1.935542011	7.575118363
multicellular organismal process (GO:0032501)	2	3.335358024
anatomical structure development (GO:0048856)	2.048176965	4.966576245
regulation of locomotion (GO:0040012)	2.055517328	7.93930216
blood vessel morphogenesis (GO:0048514)	2.204119983	4.228412519
tissue development (GO:0009888)	2.229884705	9.801342913
developmental process (GO:0032502)	2.301899454	3.756961951
regulation of cellular process (GO:0050794)	2.389339837	2.326058001
regulation of biological process (GO:0050789)	2.436518915	5.782516056
biological regulation (GO:0065007)	2.437707136	5.571865206

Supplemental Data Table 4: Genes associated with regulation of locomotion (GO: 0032879), Related to Figure 2. The 31 genes used for The Cancer Genome Atlas analysis of 112 breast cancer patients as plotted in Figure 2G.

PODXL

NOV
ROBO1
ARSB
EPPK1
MMP14
EPB41L4B
IL1R1
TIE1
FGF13
SEMA6B
FGF1
CEACAM6
DEFB1
DSP
ATOH8
ATP1A2
ATP2C2
ENNP1
F2RL3
HCAR2
IL1R1
TIE1
PTGES
KISS1
RAB3D
GPR68
RAB11FIP1
ABCA3
STOM
WNK2

Supplemental Data Table 5: Cell line culture conditions, Related to Figure 5. Media formulation for each cell line. Note the following abbreviations: Dulbecco's Modified Eagle Medium (DMEM), fetal bovine serum (FBS), penicillin/streptomycin (P/S), hEGF, and horse serum (HS).

<table>
<tr><th>Cell Line</th><th>Media</th></tr>
<tr><td>MDAMB-231</td><td rowspan="2">DMEM + 10% FBS + 1% P/S</td></tr>
<tr><td>MDAMB-468</td></tr>
</table>

BT20	
MCF-7	DMEM + 10% FBS + 1% P/S + 10 ug/mL insulin
BT549	DMEM + 10% FBS + 1% P/S + 1ug/mL insulin
SUM1315	DMEM/F-12 + 5% FBS + 1% P/S + 5ug/mL hEGF, 5ug/mL insulin
MCF10AT	Growth media: DMEM/F-12 + 5% HS + 0.5 ug/mL hydrocortisone, 20 ng/mL hEGF, 10 ug/mL Insulin, 100 ng/mL cholera toxin + 1% P/S
MCF10A-DCIS	
MCF10A	Resuspension media: DMEM/F-12 + 20% HS + 1% P/S

Supplemental Data Table 6: Patient identifier table, Related to Figure 7. Age, weight, receptor positivity, disease type, and previous treatments are reported for each patient. Patients 1-4 are not shown as were used for assay optimization. Patients 10 and 11 not shown as samples were not viable post digestion. Patients 13 and 21 did not consent to surgery, and patient 15 was on weight loss drug which differed from other patients' medication regimen, hence that patient was excluded from study. *Patient 6 received a lumpectomy on breast with TNBC and received a reduction on benign, normal tissue on contralateral breast. Patient had complete pathological response prior to treatment, so tissue treated as breast reduction. **Patient 14 had invasive ductal carcinoma in one breast, the contralateral breast was non-cancerous but underwent preventative mastectomy, so it was treated as a breast reduction. ***Patient 20 had DCIS in one breast. The contralateral breast was non-cancerous but underwent preventative mastectomy, so it was treated as a breast reduction.

**Patient Identifier†**	**Age (Years)**	**Weight (kg)**	**Receptor Positivity (TNBC, ER, HER2, PR)**	**DCIS, Invasive Cancer Type**	**Previous Treatments (specific, type, number of cycles, response)**
E005	54	77.3	N/A Breast Reduction	N/A	N/A
E006*	65	79.3	TNBC	Invasive ductal carcinoma	AC + Pembrolizumab, Pembrolizumab (200 mg in sodium chloride 0.9% 100 mL infusion), Doxorubicin (116 mg in 58 mL chemo injection), cyclophosphamide (1,160 mg in sodium chloride 0.9% 250 ml chemo infusion),

					cephalexin 500 mg, 4 cycles
E007	56	73	TNBC	DCIS	No known presurgical therapy
E008	62	74.4	ER+, PR+, HER2-	DCIS	No known presurgical therapy
E009	55	67.9	ER+, PR-, HER2 not assessed	DCIS	No known presurgical therapy
E012	76	63.2	ER+, PR-, HER2 not assessed	Invasive lobular carcinoma	No known presurgical therapy
E014**	42	74.8	ER+, PR+, HER2-	Invasive ductal carcinoma	No known presurgical therapy
E016	53	69	ER+, PR+, HER2-	Invasive mammary carcinoma	Adjuvant ddAC, adjuvant radiation, adjuvant goserelin, adjuvant anastrozole, zoledronic acid, adjuvant exemestane
E017	40	81.5	ER+, PR+, HER2-	Invasive ductal carcinoma	Unknown
E018	54	69.7	N/A Breast Reduction	N/A	N/A
E019	46	54.6	ER+, PR+, HER2-	DCIS	No known presurgical therapy

E020***	41	63.5	ER+, PR+, HER2 not assessed	DCIS	No known presurgical therapy
E022	52	72.3	ER+, PR+, HER2-	Invasive ductal carcinoma with DCIS	No known presurgical therapy
Average	53.538	70.807692			
STDEV	10.203	7.3676615			

*Patient 6 received a lumpectomy on breast with TNBC and also received a reduction on benign, normal tissue on contralateral breast. Patient had complete pathological response to prior treatment, so tissue treated as breast reduction.
**Patient 14 had invasive ductal carcinoma in one breast. Contralateral breast was non-cancerous but underwent preventative mastectomy, used as breast reduction sample.
***Patient 20 had DCIS in one breast. Contralateral breast was non-cancerous but underwent preventative mastectomy, used as breast reduction sample.
†Patients E001-E004 were used for assay optimization.
